# Identification of a core transcriptional program driving the human renal mesenchymal-to-epithelial transition

**DOI:** 10.1016/j.devcel.2024.01.011

**Published:** 2024-02-09

**Authors:** John-Poul Ng-Blichfeldt, Benjamin J. Stewart, Menna R. Clatworthy, Julie M. Williams, Katja Röper

**Affiliations:** 1 MRC-Laboratory of Molecular Biology, Francis Crick Avenue, Cambridge Biomedical Campus, Cambridge CB2 0QH, UK; 2 Molecular Immunity Unit, Department of Medicine, MRC-Laboratory of Molecular Biology, University of Cambridge, Cambridge, UK; 3 Cellular Genetics, Wellcome Sanger Institute, Hinxton, UK; 4 Cambridge Institute of Therapeutic Immunology and Infectious Diseases, University of Cambridge, Cambridge, UK; 5 Bioscience Renal, Research and Early Development, Cardiovascular, Renal and Metabolism, BioPharmaceuticals R&D, AstraZeneca, Gothenburg, Sweden

## Abstract

During kidney development, nephron epithelia arise *de novo* from fate-committed mesenchymal progenitors through a mesenchymal-to-epithelial transition (MET). Downstream of fate specification, transcriptional mechanisms that drive establishment of epithelial morphology are poorly understood. We used human iPSC-derived renal organoids, which recapitulate nephrogenesis, to investigate mechanisms controlling renal MET. Multi-ome profiling via snRNA-seq and ATAC-seq of organoids identified dynamic changes in gene expression and chromatin accessibility driven by activators and repressors throughout MET. CRISPR interference identified that paired box 8 (PAX8) is essential for initiation of MET in human renal organoids, contrary to *in vivo* mouse studies, likely by activating a cell-adhesion program. While Wnt/β-catenin signaling specifies nephron fate, we find that it must be attenuated to allow hepatocyte nuclear factor 1-beta (HNF1B) and TEA-domain (TEAD) transcription factors to drive completion of MET. These results identify the interplay between fate commitment and morphogenesis in the developing human kidney, with implications for understanding both developmental kidney diseases and aberrant epithelial plasticity following adult renal tubular injury.

## Graphical abstract

**Graphical abstract:**
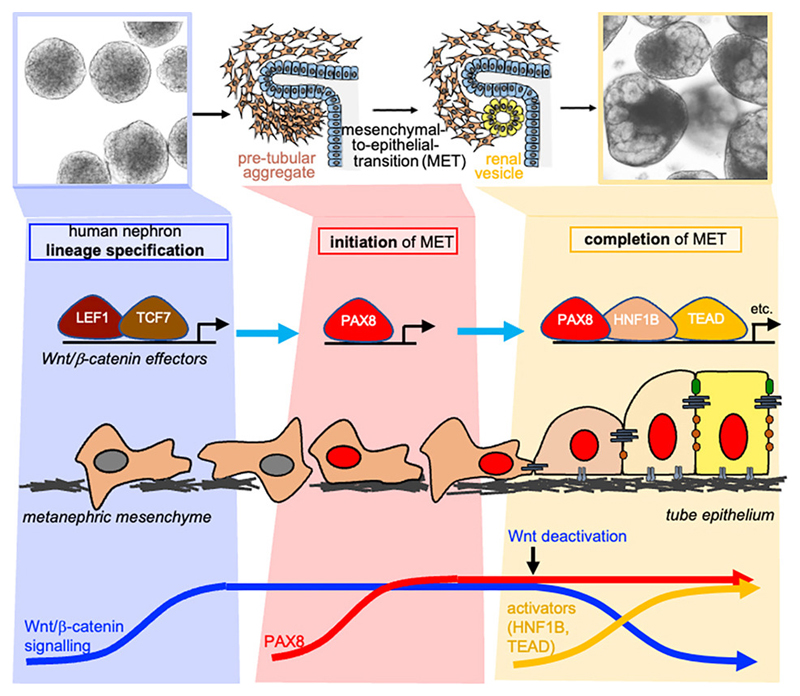

## Introduction

Emergence of complex tissues from simple tissue primordia begins with specification of tissue fate. Inductive signals, often produced by neighboring tissues, activate gene regulatory networks within competent cells that establish identity and initiate cell differentiation and morphogenesis programs. Ultimately, these lead to establishment of complex tissue shapes that enable specialized organ functions.^1^ The functional domains of most internal organs consist of epithelial sheets organized into tubular networks. Epithelial cells possess cell-scale morphological features that enable specialized tissue-scale functions, including apical-basal polarity, apical-lateral adherens junctions that provide adhesion and mechanical coupling to adjacent cells, and apical tight junctions that provide a membrane diffusion barrier and restrict paracellular flow of molecules.^2^ How tissue-specific fate programs drive establishment of epithelial morphology is poorly understood.

**Figure 1:**
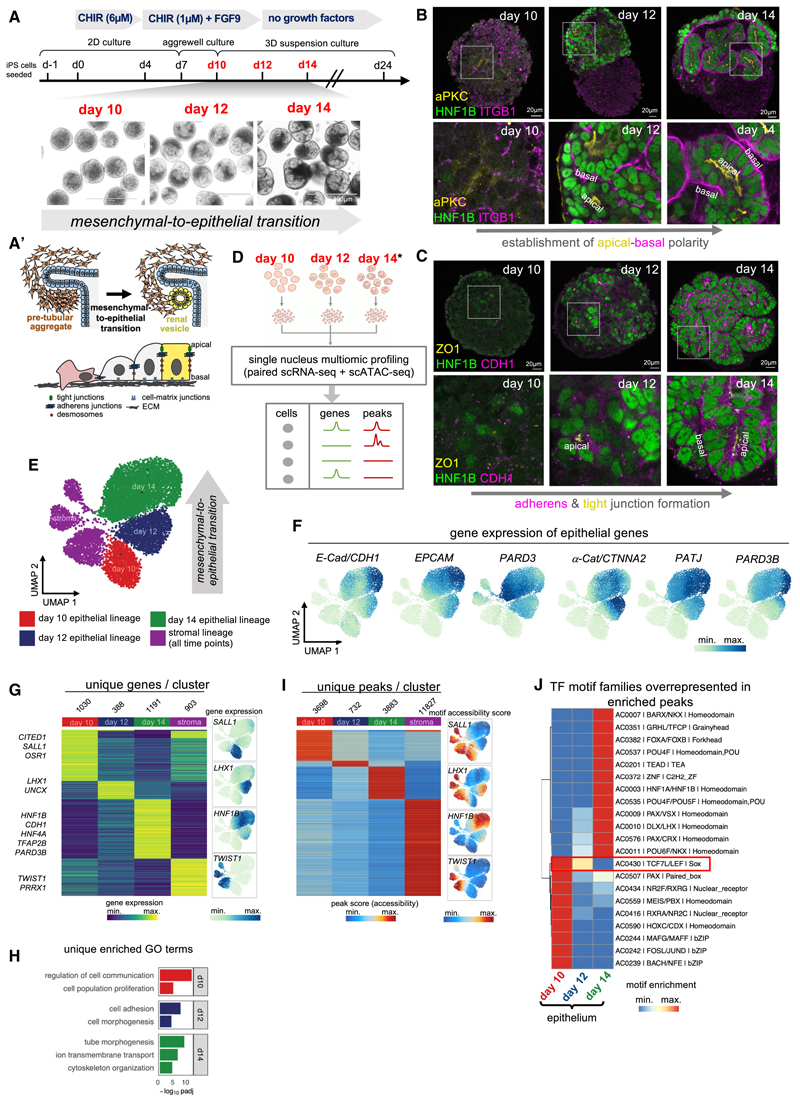
snRNA-seq and snATAC-seq profiling of human renal organoids identifies dynamic gene expression and chromatin accessibility signatures during renal MET (A) Schematic of protocol used to generate human renal organoids from iPSCs, with corresponding light microscopy images of organoids during MET (days 10–14) (protocol adapted from Kumar et al.^5^ and Takasato et al.^6^). Scale bars, 400 μm. (A^’^) Schematic of MET in the human kidney through polarization of pre-tubular aggregate cells into a renal vesicle and schematic of cellular changes during epithelial polarization. (B and C) Immunofluorescence showing expression of (B) aPKC (yellow), integrin β1 (ITGB1) (magenta), and HNF1B (green), or (C) ZO1 (yellow), E-cadherin/CDH1 (magenta), and HNF1B (green), in renal organoids over the time course of MET. Dotted white boxes indicate positions of magnification panels below, scale bars, 20 μm. (D) Schematic of renal organoid multi-ome profiling strategy for batch 2 and batch 3 individually. *For batch 3, two day 14 samples were sequenced in parallel: one treated with DMSO from days 12 to 14, and one treated with 3 μM CHIR 99021 (CHIR, a GSK3 inhibitor) from days 12 to 14 (related to Figures 7, S7, and S8). (E) UMAP representation of 9,147 cells from multi-ome batch 3 projected according to ATAC data, clustered by epithelial lineage time point, and stromal lineage (all time points combined). (F) Gene expression levels of *CDH1, EPCAM, PARD3, CTNNA2, PATJ*, and *PARD3B* overlaid on UMAP plots. (G) Heatmap of expression of marker genes for clusters in (E) determined by snRNA-seq with representative markers annotated (left, log_2_FC > 1, FDR < 0.01, two-sided Wilcoxon rank-sum test; log_2_FC, log_2_ fold change; FDR, false discovery rate). Gene expression UMAP plots of cluster-specific transcription factors (right). (H) Unique GO terms represented in enriched genes from (G), full list in Table S2. (I) Heatmap of marker peaks of accessible chromatin for clusters in (E) determined by snATAC-seq (left, log_2_FC > 1, FDR < 0.01, two-sided Wilcoxon rank-sum test). Motif accessibility UMAP plots of cluster-specific transcription factors from (G) (right). (J) Heatmap of transcription factor motif archetypes^7^ enriched in epithelial-specific peaks from (I). Annotated are archetype codes, archetypal transcription factors and DNA-binding class. Wnt/β-catenin transcriptional effectors TCF7/LEF are highlighted (red). See also Figures S1–S3.

The nephron, the functional unit of the mammalian kidney that filters the blood, is a convoluted tubular epithelial structure that arises through reciprocal inductive interactions between intermediate mesoderm-derived tissues during kidney development.^3,4^ Nephrogenesis is initiated when the ureteric bud invades the metanephric mesenchyme. Inductive signals secreted by the metanephric mesenchyme cause branching morphogenesis of the ureteric bud epithelium, while in parallel, ureteric bud-secreted signals specify nephron fate in neighboring “cap” mesenchyme cells. This leads first to condensation of the cap mesenchyme (CM) into a pre-tubular aggregate, followed by a mesenchymal-to-epithelial transition (MET; Figure 1). The MET involves a stepwise assembly of intercellular junctions and *de novo* establishment of apical-basal polarity to give rise to the renal vesicle, the first polarized epithelial precursor of the nephron. This renal vesicle subsequently elongates into the comma- and then S-shaped body and ultimately fuses with the ureteric bud tip to form the nascent nephron.^3,4^ Nephrogenesis occurs entirely prenatally and nephron endowment at birth is an important determinant of adult kidney health, with low nephron number predisposing to a range of adult kidney diseases.^8^ Therefore, a complete knowledge of mechanisms controlling nephron development is critical to better understand and prevent diseases of renal insufficiency.

The primary nephrogenic inductive cue secreted by the ureteric bud is Wnt9b,^9^ and activation of Wnt/β-catenin signaling downstream of Wnt9b is necessary and sufficient to specify nephron fate required for renal MET.^10^ However, sustained Wnt/β-catenin signaling appears to inhibit completion of renal MET,^10,11^ and Wnt/β-catenin signaling has been reported to be able to drive the opposite process, an epithelial-to-mesenchymal transition (EMT), in various instances including gastrulation and in cancer.^12–17^ Therefore, how activation of nephron fate by Wnt/β-catenin signaling leads to establishment of epithelial morphology during nephrogenesis is unclear.

Most insights into mechanisms of nephrogenesis derive from mouse models; however, recent studies highlight divergence between developing mouse and human kidneys, particularly in patterns of transcription factor (TF) expression during nephrogenesis.^18–20^ Transcriptome profiling at single-cell resolution is emerging as a powerful tool for elucidating gene expression programs of human kidney development where functional studies are not possible,^18,21–23^ however, how TFs control these programs remains largely unexplored. The recent development of a “multi-omic” approach in which transcriptomics is combined with chromatin accessibility profiling has enabled insights into how TF combinations drive gene expression programs.^24^ Moreover, the advent of human induced pluripotent stem cell (iPSC)-derived renal organoids that recapitulate nephrogenesis now allows for functional investigations into human-specific mechanisms of kidney development.^5,6,25^

To investigate TF regulation of the earliest morphogenetic changes in the developing human kidney, we applied paired transcriptome and chromatin accessibility profiling to human iPSC-derived renal organoids across the time period of renal MET combined with *in vivo/organoid* analyses and functional studies. We identify dynamic gene expression and chromatin accessibility signatures driven by transcriptional activators and repressors and identify PAX8 as a critical upstream regulator of human renal MET. Whereas it was previously shown that mice lacking PAX8 have no kidney defects,^26^ we find using CRISPR interference that PAX8 is essential to initiate MET in human renal organoids. We further find that attenuation of Wnt/β-catenin signaling is required for hepatocyte nucear factor 1-beta (HNF1B) and TEA-domain (TEAD) family TFs to drive completion of MET and maturation of the epithelial state. Our study identifies aspects of how fate commitment and morphogenesis of the early developing kidney interplay, while providing a rich resource to explore early human kidney morphogenesis and development in greater depth.

## Results

### Human iPSC-derived kidney organoids recapitulate renal MET

We set out to investigate how a key step in nephron development, the establishment of epithelial polarity preceding formation of the renal vesicle, is induced and controlled during human nephrogenesis, adapting published protocols to derive renal organoids from human iPSCs^5,6^ (Figures 1A and S1A). Analysis of epithelial polarity markers atypical protein kinase C (aPKC; apical) and Integrin beta-1 (ITGB1; basal), and epithelial junctional markers Zonula occludens-1 (ZO1; tight junctions) and E-cadherin (CDH1; adherens junctions), showed that the MET takes place between days 10 and 14 of the protocol (Figures 1A–1C). All markers progressively localized along the apical-basal axis, resembling the developing kidney *in vivo*.^27^ Organoids grown to day 24 contained recognizable renal tubular structures containing podocytes (Wilms Tumour protein 1/WT1^+^), proximal tube (LTL^+^, ECAD^+^), and distal tube (LTL^−^, ECAD^+^) (Figure S1B). Thus, human renal organoids generated with our protocol recapitulate nephrogenesis and contain expected nephron cell types.

To characterize epithelial polarization within the renal organoids in depth, we performed single-cell RNA sequencing (scRNA-seq) and single-nucleus multi-ome sequencing (paired single-nucleus RNA sequencing [snRNA-seq] and single-nucleus ATAC sequencing [snATAC-seq]) on organoids from four time points (days 10, 12, 14, and 24) (Figure 1D; for details see STAR Methods). First, scRNA-seq data and snRNA-seq data (without corresponding snATAC-data) were pooled and visualized as a uniform manifold approximation projection (UMAP) that upon coarse clustering separated into 2 clusters (Figures S1C–S1H; see STAR Methods). One cluster was enriched for *PAX8*, paired box 2 (*PAX2*), and epithelial cell-adhesion molecule (*EPCAM*) gene expression, corresponding to the nephron epithelial lineage, whereas the other cluster was enriched for paired mesoderm homeobox proten 1 (*PRRX1*), twist-related protein 1 (*TWIST1*), and collagen-alpha-2(I) chain (*COL1A2*) gene expression, corresponding to the stromal lineage (Figures S1C–S1H). The nephron epithelial lineage was re-clustered and annotated according to time point, cell-cycle phase, and expression of known nephron markers (Figures S1I–S1L),^28^ identifying a spectrum of differentiation states from nephron progenitors to differentiated nephron epithelial cells (Figure S1J), consistent with renal organoids recapitulating human nephrogenesis.

At day 10, characteristic epithelial genes were lowly expressed, consistent with cells being mesenchymal, whereas at day 14, *EPCAM*, E-cadherin/*CDH1*, and the polarity component partitioning defective 3B (*PARD3B*) were upregulated and showed maximal expression (Figure S1M), consistent with completion of MET by day 14. Tight junction components claudin 7 (*CLDN7*), claudin 3 (*CLDN3*), and the apical polarity determinant Crumbs 3 (*CRB3*) were upregulated at day 14 and further upregulated at day 24, suggesting progressive maturation of epithelial cells (Figure S1M). Expression of markers of differentiated nephron cell types, such as MAF bZIP transcription factor B (*MAFB*), hepatocyte nuclear factor 4-alpha (*HNF4A*), and transcription factor AP-2-beta (*TFAP2B*), was highest at day 24, consistent with a more fully differentiated nephron state (Figure S1N).

To investigate the relevance of renal organoid morphogenesis to human fetal kidney development *in vivo*, we analyzed our dataset alongside a previously described scRNA-seq dataset of 6 human fetal kidney samples collected between post conception weeks (pcw) 7 and 16,^21^ which as UMAP segregated into epithelial (*PAX8*^+^) and stromal (*TWIST1*^+^) lineages (Figures S2A and S2B). Projecting organoid data onto the human fetal dataset by time point identified congruence between renal organoids and fetal kidney, with differentiation into podocytes, proximal tube, and distal tube epithelial cells by day 24 (Figures S2A–S2Dʺ), indicating that renal organoids represent an experimentally tractable model system that faithfully recapitulates human renal MET *in vivo*.

### Multi-ome profiling of human renal organoids identifies dynamic gene expression and chromatin accessibility signatures during renal MET

To gain insights into transcriptional control of renal MET and early nephron morphogenesis at single-cell resolution, we interrogated the renal organoid multi-ome dataset. We focused on early time points with higher temporal resolution, namely days 10, 12, and 14, to capture the time period of renal MET (for details see STAR Methods), which upon coarse clustering separated into nephron (*PAX8*^+^) and stromal (*TWIST1*^+^) lineages (Figures 1E, S3A, and S3B). Classical epithelial genes increased within the nephron lineage from days 10 to 14, consistent with progression of epithelial polarization and MET (Figure 1F). Higher-resolution clustering identified that the nephron epithelial lineage at day 14 segregated into *WT1, HNF4A*, or *TFAP2B-*expressing cells, indicating that nephron segment identity is specified within renal organoids by day 14 concomitant with MET (Figures S3C–S3G; Table S1).

Nephron markers were dynamically expressed within the nephron lineage throughout MET. Cbp/p300-interacting transactivator 1 (*CITED1)*, spalt-like transcription factor 1 (*SALL1*), and Odd-skipped related transcription factor 1 (*OSR1*) were enriched at day 10, consistent with a mesenchymal nephron progenitor state, whereas the renal vesicle marker LIM homeobox protein 1 (*LHX1*) was enriched at day 12. The epithelial markers *HNF1B, CDH1, PARD3B*, together with *HNF4A* and *TFAP2B*, which control proximal tube and distal tube differentiation, respectively,^29,30^ were enriched at day 14, indicating that renal organoids follow the transcriptional trajectory of human nephrogenesis as observed in our and in previously published fetal human scRNA-seq datasets^28^ (Figures 1G and S2A–S2D; Table S2). Gene Ontology (GO) analysis of enriched genes further supports dynamic cell- and tissue-level morphogenetic processes driving MET within renal organoids (Figure 1H; Table S2) and suggest that expression of cell-adhesion genes (including adherens and tight junction components) precedes both junctional organization and maturation and nephron segment differentiation.

Analysis of ATAC data identified dynamic chromatin accessibility profiles throughout renal MET (Figure 1I), which were then analyzed for transcription-factor-binding motifs to identify candidate TFs driving these changes.^7^ This identified two classes of motif archetypes (Figure 1J). The first class was enriched at day 10 and decreased by day 14, which included motifs for transcription factor 7/lymphoid enhance binding factor (TCF7/LEF), indicating initial high Wnt/β-catenin signaling followed by later attenuation (Figure 1J). The second class of motif archetypes was absent at day 10 and increased by day 14, which included motifs for Grainyhead-like (GRHL) factors which regulate MET in other systems,^16^ TEAD factors, which are downstream effectors of Hippo/Yes-associated protein (YAP) signaling,^31^ and the renal vesicle stage markers HNF1A/B.^20^ These data suggest that renal MET is biphasic, with Wnt/β-catenin signaling being initially high and becoming attenuated as MET progresses, correlating with activation of additional TFs to drive completion of renal MET.

### Comparison of epithelium and stroma identifies early drivers of renal MET

**Figure 2:**
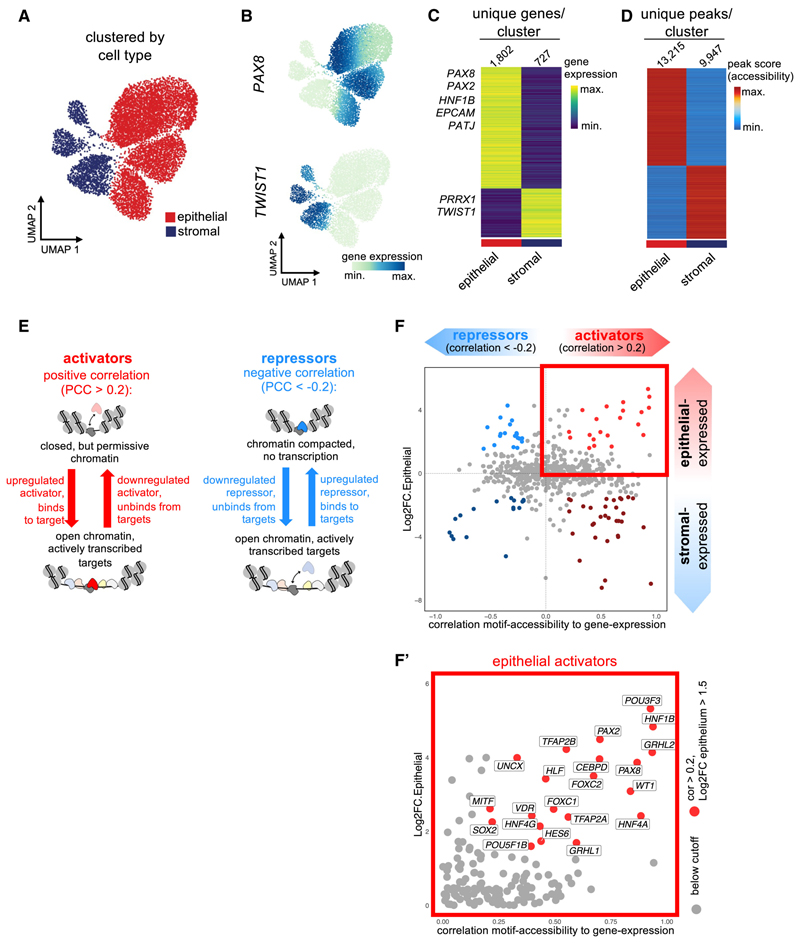
Early transcriptional activators driving renal mesenchymal-to-epithelial transition (A) UMAP of 9,147 cells from multi-ome batch 3 projected according to ATAC data, with clusters annotated according to epithelial or stromal lineage. (B) UMAP plots of multi-ome cells from batch 3, colored by *PAX8* gene expression (top) and *TWIST1* gene expression (bottom). (C) Heatmap of expression of marker genes for clusters in (A) determined by snRNA-seq with representative marker genes annotated (log_2_FC > 1, FDR < 0.01, two-sided Wilcoxon rank-sum test). (D) Heatmap of marker peaks of accessible chromatin for clusters in (A) determined by snATAC-seq (left, log_2_FC > 1, FDR < 0.01, two-sided Wilcoxon ranksum test). (E) Schematic of the principle by which correlation values, between transcription factor gene expression and corresponding motif accessibility, were used to classify transcription factors as either transcriptional repressors or activators. PCC, Pearson correlation coefficient. (F) Transcription factors plotted according to PCC of gene expression vs. corresponding motif accessibility, and log_2_FC gene expression in the epithelial lineage compared with the stromal lineage as determined by snRNA-seq. Thresholds used to color points according to principle outlined in (D): PCC > 0.2, log_2_FC epithelial lineage > 1.5 (red = activators), PCC < − 0.2, log_2_FC epithelial lineage > 1.5 (blue, repressors). (F^’^) Zoom in showing the epithelial activators identified in (F) with gene names labeled. See also Figure S4.

We next asked which TFs were central to initiating MET by investigating TFs that best defined the nephron epithelial compared with the stromal lineage (Figures 2A–2C; Table S3). Analysis of ATAC data identified differentially accessible chromatin regions between nephron (*PAX8*^+^) and stromal (*TWIST1*^+^) clusters (Figure 2D). Correlations were then derived between TF gene expression and accessibility of corresponding motifs across all cells (Figure 2E; see STAR Methods). A positive correlation implies TF binding at target loci and recruitment of epigenetic modifiers that drive chromatin opening and increased motif accessibility, a characteristic of transcriptional activators. A negative correlation implies recruitment of chromatin compactors that cause a reduction in motif accessibility, a characteristic of transcriptional repressors. This analysis identified transcriptional activators and repressors selectively expressed in either nephron epithelial or stromal lineages (Figures 2F and 2Fʹ). Corresponding analysis of a second organoid batch led to similar results (Figures S4A–S4E). Among the top epithelial activators were TFs that show segment-restricted expression within the mature nephron (e.g., *TFAP2B* and *HNF4A*), and *PAX2* and *PAX8*, which are expressed prior to acquisition of segment identity, and thus are candidate upstream regulators of renal MET.

### PAX8 expression precedes induction of MET in renal organoids and in human fetal kidney *in vivo*

**Figure 3:**
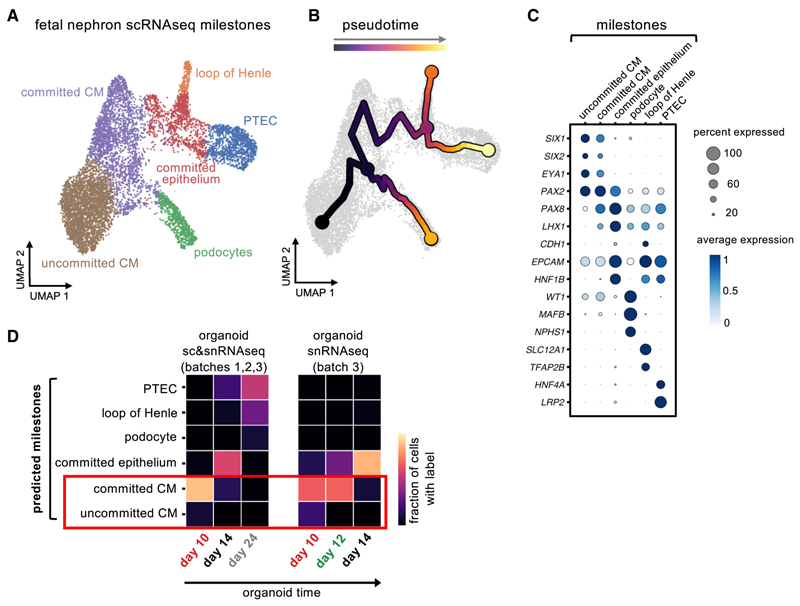
Human iPSC-derived kidney organoids show congruence with committed cap mesenchyme and subsequent epithelial derivatives in human fetal kidney *in vivo* (A) UMAP plot of 8,862 scRNA-seq cells from 6 human fetal kidney samples harvested between post conception weeks (pcw) 7 and 16, showing nephron epithelial lineage only,^21^ with developmental milestones, uncommitted cap mesenchyme (CM), committed CM, committed epithelium, podocytes, loop of Henle, proximal tubular epithelial cells (PTECs), annotated. (B) UMAP plot of 8,862 scRNA-seq cells as in (A) with the nephron pseudotime trajectory overlaid. (C) Expression of marker genes of nephron developmental stages within fetal scRNA-seq data, split across the developmental milestones highlighted in the UMAP in (A). (D) Prediction of milestone labels within organoid scRNA-seq and snRNA-seq data. Based on the milestones identified in the human fetal scRNA-seq data, the fraction of cells representing these milestones within both the pooled organoid scRNA-seq data (days 10, 14, and 24 of batches 1, 2, and 3) and the snRNA-seq data (days 10, 12, and 14 of batch 3) were analyzed and plotted as a heatmap for the different days along the protocol that were analyzed. See also Figure S2.

PAX2 and PAX8 are closely related paired box TFs that are both expressed in developing human and mouse kidneys.^20^ We first analyzed a published scRNA-seq dataset of human fetal kidney,^21^ focusing on the nephron lineage to determine *PAX2* and *PAX8* gene expression *in vivo. PAX2* was expressed in uncommitted (*SIX1*^*+*^*/SIX2*^*+*^*/Eyes absent homologue [EYA1*]^*+*^) CM, in committed CM and in subsequent committed epithelium (corresponding to renal vesicle and comma-/S-shaped body stage cells; Figures 3A–3C and S2D–S2Dʺ, consistent with published immunofluorescence analysis of PAX2 in human fetal kidney sections^20^). Notably, expression of *PAX2* in uncommitted CM indicates that PAX2 alone is insufficient to initiate renal MET. By contrast, PAX8 was absent from uncommitted CM, instead restricted to committed CM and subsequent committed epithelium (Figures 3A–3C and S2D–S2Dʺ), as previously seen in immunofluorescence for PAX8 in human fetal kidney sections,^20^ and in mice^9,32^; this pattern is highly suggestive of PAX8 having a central role in establishing the renal MET program.

Comparing transcriptomes of renal organoids and human fetal kidney showed alignment between day 10 organoids and committed CM *in vivo* (Figure 3D), consistent with organoids recapitulating induction of CM and subsequent MET. Analysis of multi-ome data showed that gene expression and motif accessibility for *PAX8* was high at day 10, with gene expression slightly reduced at day 14 (Figure S5A), mainly retained in part of a cluster identified as podocytes (Figures S3C–S3G). By contrast, *PAX2* was not highly expressed until day 12, but showed high motif accessibility at day 10 (Figure S5A). PAX2 and PAX8 share a similar DNA recognition motif,^33^ and therefore high accessibility of the shared PAX2/PAX8 motif at day 10 was likely driven by *PAX8* expression at this time point. The earlier expression of PAX8 compared with PAX2 in organoids was confirmed by immunofluorescence and qPCR (Figures S5B and S5C). The renal organoid model therefore enables the opportunity to disentangle the precise role of PAX8 from PAX2 in committed nephrogenic mesenchyme, which is usually masked *in vivo* by persistence of PAX2 from uncommitted CM and potential partial redundancy between PAX8 and PAX2.^26,34^

### PAX8 is required to initiate MET in human renal organoids

**Figure 4:**
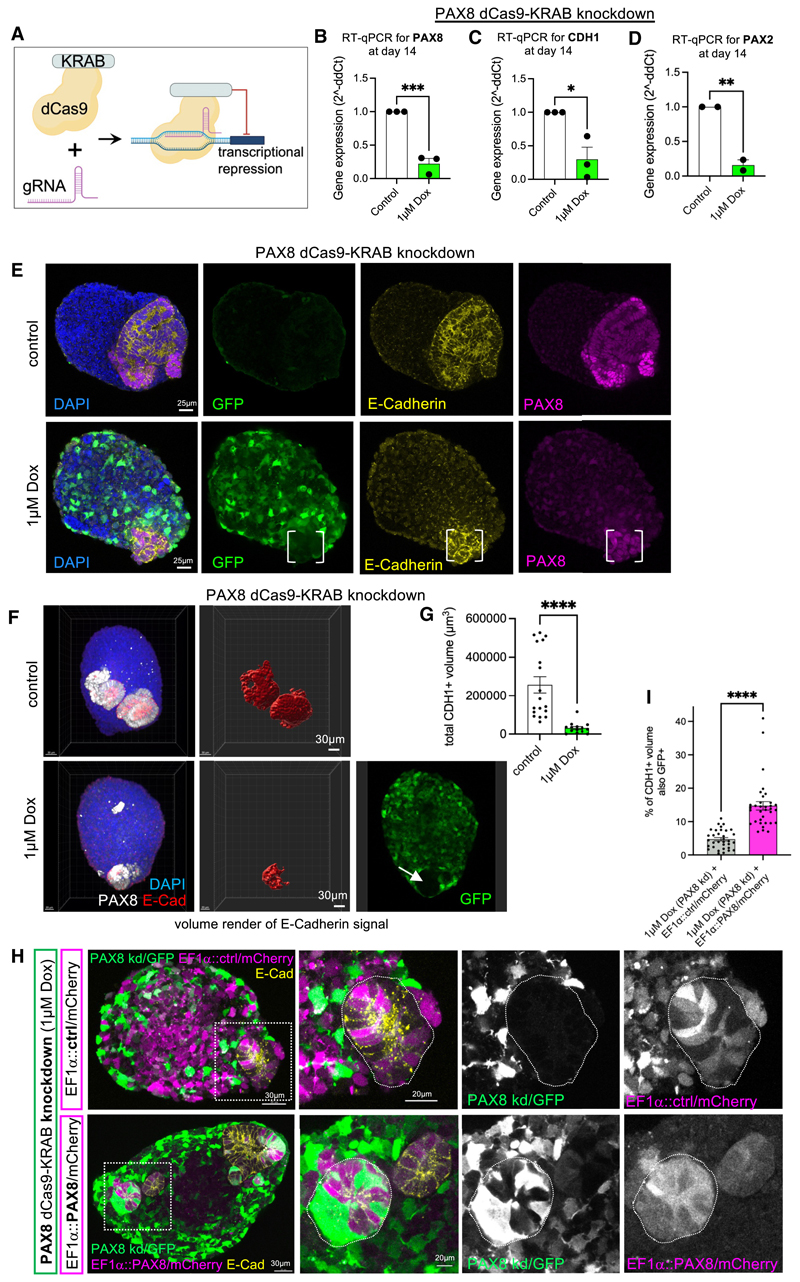
PAX8 is a critical upstream regulator of MET in human renal organoids (A) Schematic of the dCas9-KRAB CRISPR interference gene perturbation system.^35^ (B–D) RT-qPCR for (B) *PAX8*, (C) *E-cadherin/CDH1*, and (D) *PAX2* in organoids generated from PAX8-dCas9-KRAB iPSCs and harvested at day 14, either with no treatment (control, white bars) or following Dox treatment from day 4 (1 μM Dox, green bars) from 2 or 3 independent organoid batches. Dots represent data from each batch normalized to corresponding control, with bars representing mean ± SEM. Unpaired t test, *p < 0.05, **p < 0.01, ***p < 0.001, compared with corresponding control. (E) Immunofluorescence of organoids generated from PAX8-dCas9-KRAB iPSCs and harvested at day 14, either with no treatment (control, top) or following Dox treatment from day 4 (1 μM Dox, bottom), showing GFP (green), PAX8 (magenta), E-cadherin/CDH1 (yellow) with DAPI as counterstain (blue, nuclei). White brackets indicate PAX8^+^CDH1^+^ cells confined to a GFP^−^ region. Scale bars, 25 μm. (F) Immunofluorescence images showing projected z stacks of whole organoids of PAX8-dCas9-KRAB organoids at day 14, either with no treatment (control, top) or following Dox treatment from day 4 (1 μM Dox, bottom), showing PAX8 (white), E-cadherin (red), and DAPI (blue, nuclei) alongside volume render of corresponding E-cadherin/CDH1 signal (middle), and GFP expression after Dox treatment (green, right bottom). Arrow points to GFP^−^ region that contains PAX8^+^CDH1^+^ cells. Scale bars, 30 μm. (G) Quantification of volume-rendered E-cadherin/CDH1 signal in PAX8-dCas9-KRAB organoids at day 14 either with no treatment (control, white bar; n = 18 organoids across 2 independent batches) or following Dox treatment from day 4 (1 μM Dox, green bar; n = 14 organoids across 2 independent batches). Dots represent data from individual organoids, with bars representing mean ± SEM. Unpaired t test, ****p < 0.0001. (H) Rescue of MET in cells with PAX8-dCas9-KRAB-mediated PAX8 knockdown by lentiviral overexpression of PAX8. Immunofluorescence of organoids generated from PAX8-dCas9-KRAB iPSCs and harvested at day 14, following Dox treatment from day 4 (1 μM Dox, bottom), and either infected with an empty lentivirus (EF1α::ctrl/mCherry, top) or PAX8-overexpressing lentivirus (EF1α::PAX8/mCherry, bottom). GFP (green), labels PAX8-knockdown cells, magenta labels mCherry in lentivirus-infected cells (±PAX8), and E-cadherin/CDH1 is in yellow. The white box indicates regions magnified in subsequent panels, the white dotted lines mark the outline of a polarized epithelial structure. Scale bars, 20 or 30 μm as indicated. (I) Quantification of volume-rendered GFP^+^ signal as a fraction of total E-cadherin/CDH1 signal in Dox-treated PAX8-dCas9-KRAB organoids at day 14 either infected with an empty lentivirus (EF1α::ctrl/mCherry, gray bar; n = 33 organoids across 2 independent batches) or PAX8-overexpressing lentivirus (EF1α::PAX8/mCherry, magenta bar; n = 34 organoids across 2 independent batches). Dots represent data from individual organoids, with bars representing mean ± SEM. Unpaired t test, ****p < 0.0001. See also Figure S5.

The above data support the hypothesis that PAX8 is a critical upstream regulator of human renal MET. To test this, we engineered iPSCs using dead-Cas9-KRAB-based CRISPR interference (Figure 4A; Tang et al.^35^) to suppress *PAX8* gene expression under the control of doxycycline (Dox), with cytoplasmic GFP as a readout for the response to Dox (this cell line is here-after referred to as PAX8-dCas9-KRAB). As the *PAX8* gene was expressed from day 6 in the organoid protocol (Figure S5C), differentiating PAX8-dCas9-KRAB iPSCs were treated with 1 μM Dox from day 4, which led to ~80% knockdown of *PAX8* gene expression at day 14 compared with untreated control organoids (***p < 0.001; Figure 4B). *PAX8* knockdown led to a significant corresponding reduction in *CDH1* gene expression at day 14 (~80%, *p < 0.05; Figure 4C), and a significant reduction in *PAX2* expression (**p < 0.01; Figure 4D), consistent with PAX2 being downstream of PAX8 in renal organoids. Control organoids at day 14 did not express GFP, indicating lack of knockdown cassette activity, and contained PAX8^+^ cells that also expressed E-cadherin as we observed previously (Figures 4E and 4F). By contrast, Dox-treated PAX8-dCas9-KRAB organoids at day 14 showed heterogeneous GFP expression with reduced PAX8 staining. Cells that retained PAX8 also lacked GFP and thus were not responding to Dox (Figures 4E and 4F). In these organoids, E-cadherin expression was mostly depleted, and where present, was confined to the PAX8^+^ GFP-lacking cells (Figures 4E and 4F). Quantification identified ~80% reduction in E-cadherin signal volume and hence in the degree of epithelialization after PAX8-knockdown compared with control organoids (****p < 0.0001; Figures 4F and 4G). Reduced epithelialization upon PAX8 knockdown was confirmed in an independent iPSC line with a distinct gRNA sequence to target *PAX8* (Figure S5D).

To confirm specificity of the *PAX8* knockdown, we tested whether lentiviral-mediated PAX8 re-expression could rescue epithelialization in knockdown cells. PAX8-dCas9-KRAB organoids, treated with Dox to induce knockdown, were exposed at day 7 to lentiviruses harboring either a PAX8 overexpression cassette with cytoplasmic mCherry as a readout for successful transduction (Figure S5E), or a control mCherry-only overexpression cassette (Figure 4H). *PAX8*-knockdown organoids infected with control lentivirus showed widespread mCherry expression, indicating efficient uptake and expression of the lentiviral cassette. The small E-cadherin^+^ vesicles present expressed mCherry but lacked GFP expression, confirming that *PAX8*-knockdown cells failed to undergo MET (Figure 4H). By contrast, *PAX8*-knockdown organoids infected with PAX8-expressing lentivirus contained E-cadherin^+^ vesicles with cells co-expressing GFP and mCherry, indicating that PAX8 re-expression could rescue MET in *PAX8* knockdown cells (Figure 4H), confirming the critical role for PAX8 in human renal MET. Quantification showed a significant increase in the proportion of E-cadherin^+^ vesicles that was also GFP^+^ following PAX8 re-expression (Figure 4I). Of note, mCherry^+^ PAX8-overexpressing cells were typically observed within and not outside of E-cadherin^+^ vesicles, suggesting that PAX8 overexpression was sufficient to drive MET in stromal cells (Figures 4H and S5E).

We next assessed the role of PAX2 in human renal MET by engineering iPSCs that allowed Dox-dependent suppression of PAX2 expression (referred to as PAX2-dCas9-KRAB; Figures S5F and S5G). When differentiated, PAX2-dCas9-KRAB iPSCs treated with 1μM Dox from day 4 resulted in ~80% knockdown of *PAX2* gene expression at day 14 compared with untreated control organoids (***p < 0.001, unpaired t test; Figure S5G). Control organoids at day 14 lacked GFP expression and contained tubular structures positive for PAX2 and E-cadherin (Figure S5F). Dox-treated organoids expressed GFP heterogeneously, containing tubular structures positive for GFP and PAX2 in a mutually exclusive pattern, indicating that GFP^+^ cells successfully knocked down PAX2 (Figure S5F). GFP-expressing *PAX2*-knockdown cells expressed E-cadherin and contributed to tubular structures, indicating that PAX2 was dispensable for generation of nephron epithelium from human iPSCs, in accordance with a previous report.^36^

Our analyses in human renal organoids thus identify a hitherto unappreciated central role for PAX8 in initiating MET during human kidney development that, in contrast to mouse kidney development *in vivo*,^26,34^ cannot be compensated for by PAX2 in human renal organoids.

### PAX8 appears to activate a cell-adhesion gene program to initiate MET

**Figure 5:**
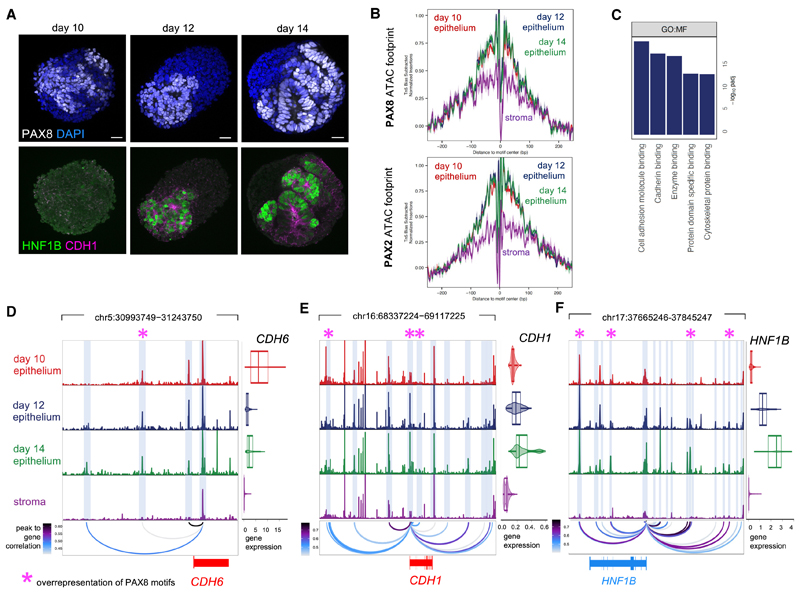
PAX8 is predicted to activate a cell-adhesion gene expression program to initiate MET in human renal organoids (A) Immunofluorescence analysis of organoids showing PAX8 (white, top) with DAPI as counterstain (blue, nuclei), with corresponding expression of HNF1B and E-cadherin/CDH1 (green and magenta, respectively, bottom) at days 10, 12, and 14. Scale bars, 25 μm. (B) Footprint analysis of PAX8 and PAX2 motifs using cluster annotations from (Figure 1E). (C) GO terms overrepresented in the predicted PAX8 regulon (full list in Table S4). (D–F) Chromatin accessibility tracks for (D) *CDH6*, (E) *E-cadherin/CDH1*, and (F) *HNF1B* loci, split according to clusters annotated in Figure 1E, epithelium days 10, 12, 14, and stroma (all time points combined), with peak to gene links highlighted. Violin plots of gene expression determined by snRNA-seq (box center line, median; limits, upper and lower quartiles; whiskers, 1.5× interquartile range). Purple asterisks indicate overrepresentation of PAX8 motifs as determined by analysis with HOMER (see STAR Methods). See also Figure S5.

We next investigated the genetic program activated downstream of PAX8 that drives MET. In day 10 organoids, PAX8 localized to coalescing cells analogous to the pre-tubular aggregate; moreover, PAX8^+^ cells selectively expressed HNF1B and E-cadherin at day 12 (Figure 5A). Footprint analysis of ATAC data showed occupancy at PAX8/PAX2 motifs in the nephron lineage at all time points, but not in stroma (Figure 5B), suggesting that PAX8 drives target gene expression throughout MET. Next, a putative PAX8 regulon was determined by analysis of peak to gene links coupled with motif analysis, which allows *in silico* predictions of target genes (Figure S5H; Table S4). GO analysis of the putative PAX8 regulon identified enrichment for “cell adhesion” and “cadherin binding” (Figure 5C; Table S4). Interestingly, the putative PAX8 regulon contained Cadherin-6 (*CDH6*), one of the earliest cadherins expressed in the mouse condensed mesenchyme *in vivo*^37^; *CDH6* was expressed in human renal organoids at day 10 prior to other cadherins (Figures S5I and S5J). Examination of the *CDH6* locus identified 4 accessible peaks linked to *CDH6* (Pearson correlation coefficient [PCC] < 0.4, false discovery rate [FDR] < 0.0001; Figure 5D). One peak 86 kb upstream of the *CDH6* transcription start site (TSS), which was accessible in the nephron lineage throughout MET, but not in stroma, showed enrichment for PAX8 motifs, suggesting that PAX8 may drive *CDH6* expression by directly binding to this genomic region (Figure 5D). In support of this, PAX8^+^ cells co-expressed CDH6 in day 10 organoids (Figure S5J).

*CDH1* and *HNF1B* were also contained within the putative PAX8 regulon and therefore potentially regulated by PAX8, consistent with co-expression of E-cadherin and HNF1B in PAX8^+^ cells in day 12 organoids (Figure 5A). Analysis of the *CDH1* locus identified 25 peaks significantly linked to *CDH1* (PCC < 0.4, FDR < 0.0001; Figure 5E), including two peaks corresponding to enhancers within the second intron of *CDH1*.^38^ PAX8 motifs were overrepresented in three *CDH1*-linked peaks. Analysis of the *HNF1B* locus identified 24 peaks significantly linked to *HNF1B* (PCC < 0.4, FDR < 0.0001; Figure 5F); PAX8 motifs were overrepresented in 3 of these peaks.

We therefore propose that PAX8 is a critical upstream regulator that initiates MET in human renal development, likely directly downstream of Wnt/β-catenin signaling,^39^ and that PAX8 could drive human renal MET by directly activating expression of genes that establish epithelial polarity and intercellular junctions. Altogether, these data help to explain how establishment of the nephron fate program drives the emergence of complex tissue morphology during human kidney development.

### A combination of transcriptional activators and repressors drives human renal MET

**Figure 6:**
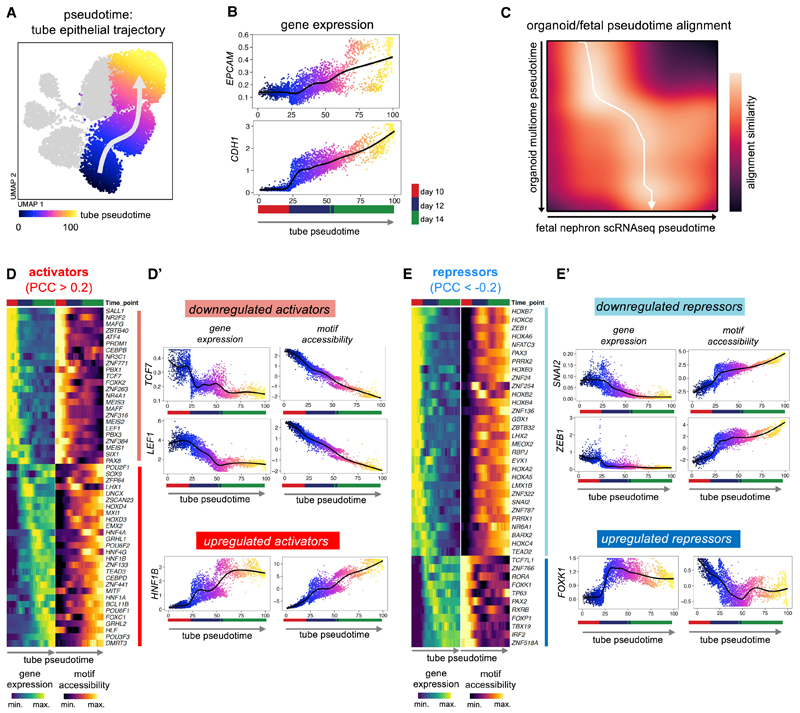
Dynamic activity of transcriptional activators and repressors drives renal MET (A) UMAP plot of 9,147 multi-ome cells from batch 3 colored according to tube epithelial pseudotime, as computed in ArchR.^24^ (B) Cells from batch 3 plotted according to tube epithelial pseudotime as in (A), and gene expression of *EPCAM* and *E-cadherin/CDH1*, with time points indicated below. (C) Alignment of organoid tube epithelial pseudotime with fetal nephron pseudotime. The arrow indicates the path through the minima of the cost matrix where the trajectories best align. (D) Heatmaps (left) of gene expression and corresponding motif accessibility along tube epithelial pseudotime for transcriptional activators (PCC gene expression vs. motif accessibility along pseudotime > 0.2). Plots (right) of gene expression and motif accessibility of example downregulated activators *TCF7* and *LEF1*, and an example upregulated activator *HNF1B*, along tube epithelial pseudotime. (E) Heatmaps (left) of gene expression and corresponding motif accessibility along tube epithelial pseudotime for transcriptional repressors (PCC gene expression vs. motif accessibility along pseudotime < −0.2). Plots (right) of gene expression and motif accessibility of example downregulated repressors *SNAI2* and *ZEB1*, and an example upregulated repressor *FOXK1*, along tube epithelial pseudotime. See also Figure S6.

We next interrogated the multi-ome dataset to identify TFs regulating the completion of polarization and epithelial maturation during MET. We used pseudotime analysis to infer a trajectory representing the emergence of renal tubule epithelial cells via MET in kidney organoids (Figure 6A), with expression of epithelial genes increasing accordingly along pseudotime (Figure 6B). Comparison with fetal nephron pseudotime showed that the organoid MET pseudotime trajectory aligned with the transition from committed CM to committed epithelium, and thus corresponded to the period of MET *in vivo* (Figures 3D and 6C). By correlating TF gene expression and motif accessibility along pseudotime, 52 activators and 41 repressors were identified (FDR < 0.001), identifying a temporal sequence of activator and repressor function during renal MET (Figures 6D and 6E).

Two classes of activators were observed. The first showed downregulated gene expression during MET; this included *TCF7* and *LEF1*, consistent with attenuation of Wnt/β-catenin signaling throughout MET (Figure 6D′). Interestingly, *LEF1* expression and motif accessibility decreased at day 12 compared with day 10 despite continued presence of 1 μM CHIR in the organoid protocol until day 12 (Figure 6D′ top), suggesting a mechanism to attenuate Wnt/ β-catenin signaling downstream of glycogen-synthase kinase 3 (GSK3). The second class of activators were upregulated during MET; this included *HNF1B* (Figure 6D′ bottom), consistent with our previous ATAC data (Figure 1J) and in agreement with the well-established role of HFN1B in renal development.^40^

Two classes of repressors were observed. One class showed decreased gene expression and increased motif accessibility during MET, which included the EMT-associated TFs (EMT-TFs) Snail-family transcriptional repressor 2 (*SNAI2*) and zinc finger E-box binding homeodomain 1 (*ZEB1*) (Figure 6E′ top). The second class of repressors showed upregulated gene expression with decreased motif accessibility during MET (Figure 6E′ bottom), which included the forkhead family TF *FOXK1*, which was described as a transcriptional repressor in myoblasts.^41^ Corresponding analysis of a second batch identified similar trajectories for all these TFs during MET (Figures S6A–S6C).

Altogether, these analyses identify that attenuation of Wnt/β-catenin signaling during MET is associated with dynamic activity of specific activators and repressors that are likely to drive the continuation and completion of human renal MET, and whose individual roles can now be probed using the organoid system.

### Attenuated Wnt/β-catenin signaling permits completion of renal MET

**Figure 7:**
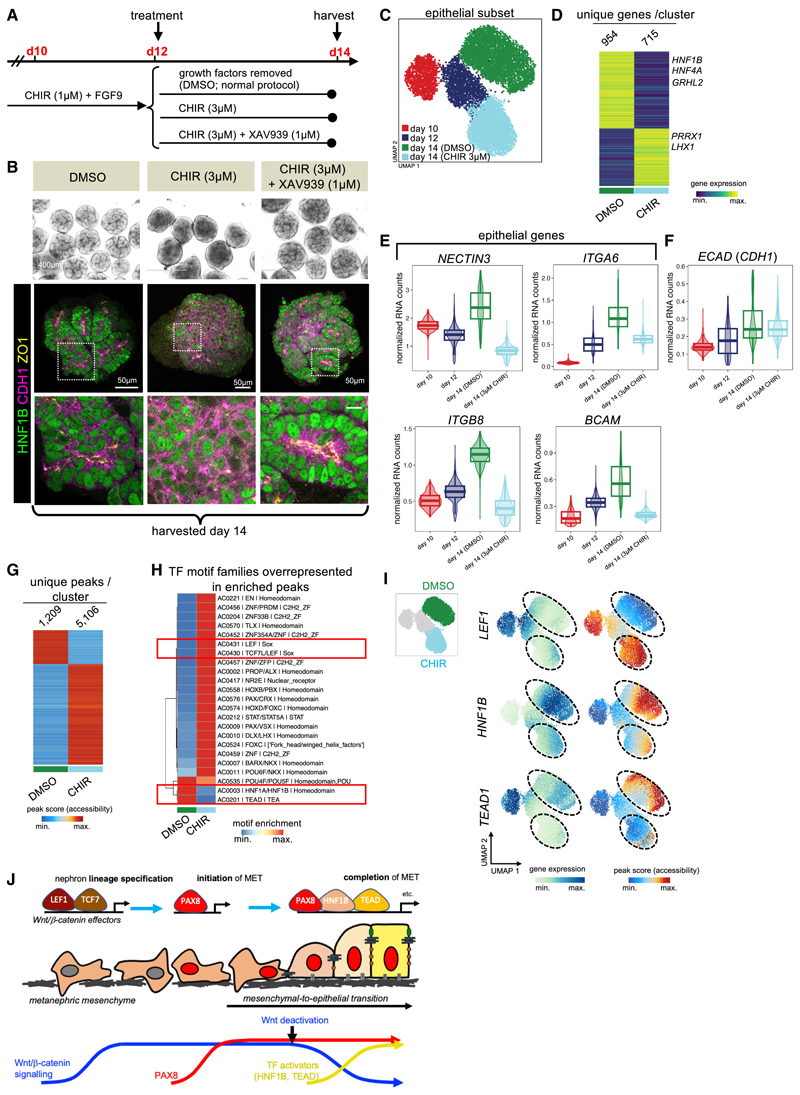
Attenuation of Wnt/β-catenin signaling allows completion of MET via HNF1B and TEAD (A) Schematic of experimental design to test the effect of persistent Wnt/β-catenin pathway activation on completion of renal MET. (B) Organoids harvested at day 14 following treatments indicated in (A), with light microscopy images (top, scale bar 400 μm), and immunofluorescence images showing expression of HNF1B (green), E-cadherin/CDH1 (magenta), and ZO1 (yellow). Scale bars, 50 μm. (C) UMAP of cells from multi-ome batch 3 containing additional CHIR-treated group. (D) Heatmap of marker genes expression levels for DMSO-treated or CHIR-treated epithelium at day 14 determined by snRNA-seq with representative markers annotated (log_2_FC > 1, FDR < 0.01, two-sided Wilcoxon rank-sum test). (F and G) Gene expression violin plots of (E) *NECTIN3, ITGA6, ITGB8*, and *BCAM*, and (F) *E-cadherin*/*CDH1*, according to clusters annotated in (C). Box center line, median; limits, upper and lower quartiles; whiskers, 1.5× interquartile range. (G) Heatmap of marker peaks of chromatin accessibility for DMSO-treated or CHIR-treated epithelium at day 14 determined by snATAC-seq (left, log_2_FC > 1, FDR < 0.01, two-sided Wilcoxon rank-sum test). (H) Heatmap of transcription factor motif archetypes^7^ overrepresented in differentially accessible peaks from (G). Annotated are archetype codes, archetypal transcription factors and DNA-binding class. Highlighted in red are Wnt/β-catenin transcriptional effectors LEF1 and TCF7/LEF, and HNF1A/HNF1B and TEAD family transcription factor motif archetypes. (I) UMAP plots highlighting DMSO- and CHIR-treated epithelium at day 14 (top), with UMAPs corresponding to gene expression (left) and motif accessibility (right) for *LEF1, HNF1B*, and *TEAD*. (J) Our proposed model of the transcriptional control of morphogenetic events during human renal MET. See also Figures S7 and S8.

Wnt/β-catenin signaling specifies nephron fate, a critical part of which, as we have shown, is PAX8-driven initiation of MET. However, it was reported that sustained Wnt/β-catenin signaling blocks completion of renal MET,^10,11^ suggesting that persistent Wnt/β-catenin signaling could inhibit TFs needed to properly organize epithelial polarity and junctions. Therefore, we investigated how completion of renal MET and epithelial maturation are regulated by pharmacologically sustaining Wnt/β-catenin signaling in organoids until day 14. As *LEF1* gene expression and motif accessibility were decreased at day 12 despite the presence of 1 μM CHIR (Figures 6D and 6D′), we used 3 μM CHIR to ensure sustained Wnt/β-catenin signaling beyond day 12 (Figure 7A). Organoids at day 12 (when MET has initiated but adherens and tight junctions remain disorganized; Figures 1B, 1C, and S7A) were treated with DMSO as a control, with 3μM CHIR, or with 3 μM CHIR plus the tankyrase inhibitor XAV939 (1μM) to counteract the effects of CHIR and rescue any potential phenotype^42^; organoids were then cultured until day 14 (Figure 7A). Control organoids at day 14 contained HNF1B^+^ tubular epithelium with polarized localization of E-cadherin and ZO1 (Figures 7B and S7B). Strikingly, organoids in which Wnt/β-catenin signaling was sustained lacked clear tubular structures and, instead, showed diffuse E-cadherin and reduced HNF1B staining. ZO1 staining was reduced and instead observed in sparse foci, indicating a failure of proper epithelial junction organization (Figures 7B and S7B), similar in appearance to control organoids at day 12 (Figure S7A). Addition of XAV939 rescued tubular morphology and resulted in organoids indistinguishable from control organoids (Figures 7B and S7B).

Thus, sustained Wnt/β-catenin signaling blocked completion of MET in renal organoids. Notably, this effect was reversible, as a period of CHIR washout from days 14 to 17 produced organoids that contained HNF1B^+^ tubular structures with normal localization of E-cadherin and ZO1 similar to control organoids at day 17 (Figures S7C and S7D). Therefore, transcriptional factors that drive proper epithelial junctional organization and maturation are activated upon attenuation of Wnt/β-catenin signaling. To investigate this further, a subset of organoids were treated from days 12 to 14 with 3 μM CHIR and analyzed by multi-ome sequencing (Figures S7E and S7F). The nephron epithelial lineage (*PAX8*^*+*^, *EPCAM*^+^, *TWIST1*^−^, Figure S7G) was re-clustered, resulting in 4 clusters corresponding to days 10, 12, and 14 treated with either DMSO or with 3 μM CHIR from days 12 to 14 (Figure 7C).

Treatment with 3 μM CHIR from days 12 to 14 sustained Wnt/β-catenin signaling as shown by persistent expression of the Wnt target gene *AXIN2* and increased accessibility of TCF7 motifs (Figure S8A). Sustained CHIR prevented downregulation of nephrogenesis markers *LHX1* and homeobox protein unc-4 homologue (*UNCX*) that would normally occur between days 12 and 14, suggesting a delay in the normal progression of nephrogenesis (Figure S8B). According to GO analysis, sustained CHIR treatment caused downregulation of genes involved in cell adhesion and ion transport, suggesting defects in epithelial polarization and nephron differentiation (Figures 7D–7F and S8C; Table S5). Sustained CHIR treatment drove downregulation of the cell-adhesion molecule *NECTIN3*, previously shown to help establish epithelial polarity during normal kidney development,^43^ and downregulation of integrin alpha-6 (*ITGA6*), integrin beta-8 (*ITGB8*), and basal cell adhesion molecule (*BCAM*), which are basolaterally localized adhesion molecules; their downregulation could disrupt polarity establishment (Figure 7E). Gene expression for *E-cadherin/CDH1* was unchanged (Figure 7F). Sustained Wnt/β-catenin did not directly repress *E-cadherin/CDH1* expression (Figure 7F), but instead appeared to dysregulate other adhesion molecules to impair establishment and maintenance of epithelial polarity.

Differentially accessible chromatin regions upon CHIR treatment (Figure 7G) were analyzed for TF motifs to identify candidate TFs regulating epithelial morphology.^7^ TCF7/LEF1 motifs were enriched in CHIR-treated epithelium, confirming sustained Wnt/β-catenin signaling (Figure 7H), whereas HNF1A/B and TEAD motif archetypes were selectively depleted following CHIR treatment (Figures 7H and 7I). These data suggest that Wnt/β-catenin signaling may normally repress the activities of HNF1A/B and TEAD and imply that attenuation of Wnt/β-catenin signaling during MET may activate them to drive organization and maintenance of epithelial polarity and junctions (Figure 7J).

Further analysis of multi-ome data identified loss of proximal and gain of distal tubule identity upon sustained Wnt/β-catenin signaling (Figures S8D–S8G). Notably, CHIR treatment caused a complete loss of motif accessibility for the Notch signaling effector recombination-signal-binding protein for immunoglobulin kappa J region (RBPJ) (Figures S8G and S8H). Previous studies implicated Notch signaling in proximal fate differentiation during nephron segmentation^44^; accordingly, CHIR treatment downregulated the Notch pathway genes *NOTCH2* and Delta-like canonical Notch ligand 1 (*DLL1*), consistent with Wnt/β-catenin signaling inhibiting proximal differentiation via Notch inhibition (Figure S8I).

We conclude that attenuation of Wnt/β-catenin signaling appears crucial to allow HNF1A/B and TEAD family TFs to drive completion of MET, through their effects on the proper organization and maintenance of epithelial junctions. However, the additional role of Wnt/β-catenin signaling in proximal tube differentiation means this attenuation must be carefully balanced for normal nephrogenesis to proceed.

## Discussion

How the genome encodes form and hence function is still a major unresolved question in biology. Using human iPSC-derived renal organoids as a human developmental model, we have mapped TF dynamics and resultant gene expression programs at single-cell resolution during the earliest acquisition of form and shape in human nephrogenesis, the renal MET, elucidating the genetic cascade leading from fate commitment to morphogenesis (Figure 7J).

Pairing of transcriptome and chromatin accessibility profiling enables additional insights compared with the use of either approach alone, one powerful example being derivation of correlations between TF gene expression and genome-wide accessibility of corresponding motifs. Using this approach, we identified numerous transcriptional activators and repressors potentially driving MET. Of note, we observed downregulation of the EMT-TFs SNAI2 (Slug), in agreement with previous studies in mouse,^45^ and also of ZEB1; both SNAI2 and ZEB1 can directly bind to and repress epithelial genes including *E-cadherin/CDH1*,^16,46,47^ and therefore their downregulation may allow progression and completion of MET. Our comprehensive multi-ome dataset identified additional activators and repressors that may have a key role in MET, opening up many avenues for future work.

Our study strongly suggests that PAX8 directly drives MET during human kidney development. To date, investigation into the precise role of PAX8 in renal MET has been hampered by partial redundancy with PAX2 *in vivo*.^26,34^ Moreover, PAX8 is expressed *in vivo* not only in committed CM but also in collecting duct and in earlier transient pro- and meso-nephric structures during renal development.^34^ Therefore, while *PAX8* mutations are associated with kidney defects in humans (albeit infrequently; Meeus et al.^48^ and Carvalho et al.^49^), this could reflect a role in these other renal structures. The renal organoid system we used produces no ureteric bud derivatives and appears to bypass uncommitted CM to instead recapitulate committed CM and subsequent MET where PAX8 is expressed *in vivo* and thus provides the unique opportunity to study the precise role of PAX8 in renal MET. Furthermore, our multi-ome approach enables unbiased identification of putative TF regulons. With this approach, we identified genes potentially regulated by PAX8, which by GO analysis were enriched for cell-adhesion genes. In particular, putative activation of the key adhesion genes *CDH6* and *E-cadherin/CDH1* by PAX8 helps to explain how PAX8 could drive polarization and initiate MET. The survey of putative PAX8-regulated genes presented here is likely to be of interest given the emerging evidence of PAX8-dependency in the development of adult human cancers including renal cell clear cell carcinoma and ovarian cancer^50,51^; however, we note that these *in silico* analyses are predictive and further studies directly probing PAX8 chromatin binding are required to better understand the PAX8 target gene repertoire. All peak to gene links identified in our study are provided in Table S6 as a resource to further explore predicted gene programs controlled by TFs driving human renal MET.

Our functional data diverge from previous studies where mice with germline *Pax8*-null mutations had no apparent kidney phenotype, attributed to compensation by Pax2 *in vivo*.^26,34^ PAX8 and PAX2, together with PAX5, comprise a PAX family subgroup and share a conserved DNA-binding paired domain, a partial homeodomain, and an octapeptide domain and have partially redundant functions *in vivo*.^52^ Our observation from scRNA-seq that *PAX2* is expressed in uncommitted CM in human fetal kidney *in vivo* and persists upon commitment and sub-sequent MET, in agreement with published immunofluorescence analyses,^20^ explains how PAX2 may compensate *in vivo* for loss of PAX8. Nonetheless, the presence of PAX2 in uncommitted CM with no apparent change at the onset of MET argues that PAX2 is unlikely to be directly driving renal MET as previously suggested.^53,54^ PAX2 can form a complex with HOX paralogs and EYA1 to directly drive *Six2* expression, implicating PAX2 in the control of nephron progenitor self-renewal as opposed to commitment.^55^ Moreover, selective depletion of *Pax2* in *Six2*+ cells in mice caused ectopic transdifferentiation into renal stroma,^56^ suggesting that Pax2 may function to repress an interstitial fate within uncommitted CM. We speculate that specific co-factors are expressed upon commitment of CM together with PAX8 that allow PAX8 to activate the MET program, while permitting these potentially divergent functions of PAX2. The multi-ome data presented herein provide a resource to aid future studies aimed at elucidating co-factors recruited by PAX8 and PAX2 to mediate gene regulation during renal MET.

It was known from studies by Park et al. that Wnt/β-catenin signaling, though crucial to specify nephron fate, must act transiently for successful MET,^10,11^ but the reason underlying this was unknown. Applying multi-ome profiling to the renal organoid system allowed us to address this question, by identifying global TF dynamics during MET as Wnt/β-catenin signaling is attenuated, and enabling investigation into the impact of sustained Wnt/β-catenin signaling with CHIR. Wnt/β-catenin signaling has a close relationship with the epithelial state in part owing to the dual role of β-catenin at adherens junctions, and as a transcriptional co-activator for TCF/LEF.^57^ One possibility is that Wnt/β-catenin activation sequesters β-catenin in the nucleus, preventing recruitment to adherens junctions and thus inhibiting their proper organization. However, our finding that sustained Wnt/β-catenin signaling caused downregulation of genes involved in cell adhesion (e.g., *NECTIN3, ITGA6, ITGB8*, and *BCAM*) argues that the Wnt-dependent transcriptional program is broadly antagonistic to renal MET.

Our finding that Wnt/β-catenin signaling becomes attenuated despite the continued presence of CHIR in the culture media until day 12 suggests an intrinsic mechanism to limit Wnt/β-catenin signaling during MET. We found that the Hippo-YAP/TAZ pathway effector TEAD^31^ may mediate completion of renal MET, raising the intriguing possibility that biomechanical feedback could inhibit Wnt signaling. Increased tension at epithelial adherens junctions can sequester the serine/threonine kinases Lats1/2, thereby promoting YAP/TAZ accumulation and signaling.^58–60^ In turn, YAP/TAZ can directly promote β-catenin degradation to inhibit Wnt/β-catenin signaling,^61^ together suggesting that Wnt signaling could be attenuated in response to tension establishment at maturing junctions, as would occur during renal MET. YAP/TEAD signaling itself must be carefully controlled for proper nephrogenesis, as deletion of *Lats1/2* in Six2^+^ CM cells in mice led to loss of nephrons, with mutant cells instead expressing mesenchymal markers.^62^ Mechanisms leading to TEAD activation during renal MET remain to be determined, but one possibility could involve direct interactions between TEAD and PAX8, as has been observed in Müllerian epithelial cells.^63^ Another possibility suggested by our study could involve interaction between TEAD and HNF1B, which could co-operate to drive maturation of the renal epithelial state. Accordingly, studies of mutant mice with kidney-specific deletion of HNF1B showed that the initial epithelial polarization was unaffected by HNF1B loss, with defects instead observed at comma- and S-shaped body stages, consistent with a role for HNF1B in the completion of epithelial maturation and maintenance of the epithelial state but not the initial MET itself.^64^ HNF1B has also been reported to antagonize Wnt/β-catenin signaling by both directly repressing *LEF1* gene expression and by competitively binding and antagonizing TCF/LEF1-target genes.^65,66^ Increased expression of HNF1B, for example, by direct activation by PAX8 as we observed, could thus provide another possible feedback mechanism to limit Wnt/β-catenin signaling during renal MET.

Our study sheds light on the interplay between fate commitment and morphogenesis within the developing human kidney and offers a rich resource for the community to explore mechanisms controlling early nephron development in greater depth. Our study is therefore likely to have implications for our understanding of developmental kidney disease but also aberrant epithelial plasticity incurred by acute injury to renal tubular epithelium in adulthood. Acute kidney injury is thought to re-engage developmental processes, particularly activation of Wnt/β-catenin signaling leading to partial EMT, cellular responses which contribute to tubular repair but can also pre-dispose to chronic kidney disease.^67^ A more complete picture of early events in renal morphogenesis as presented here may therefore help toward a better understanding of how to modulate the response to acute kidney injury for successful kidney regenerative strategies.

### Limitations of the study

The human renal organoid model employed here is a reductionist system but nonetheless captures the early stages of human nephrogenesis well, as our study illustrates. What the system does not recapitulate is the overall developing three-dimensional tissue structure, which in the kidney is instructed by the branching morphogenesis of the collecting duct system, the ureteric bud, that together with endothelial cells is not present in this organoid system. Nonetheless, the local tissue geometry of renal vesicle and early expanding tube are well recapitulated. Furthermore, single-cell and single-nucleus transcriptomic analyses by their nature cause loss of spatial information; further studies using recently developed spatial transcriptomic platforms will help to delineate spatial localization of regulators during renal MET.

## Star*Methods

Detailed methods are provided in the online version of this paper and include the following:

- KEY RESOURCES TABLE
- RESOURCE AVAILABILITY
  - Lead contact
  - Materials availability
  - Data and code availability
- EXPERIMENTAL METHOD AND STUDY PARTICIPANT DETAILS
  - Cell lines
- METHOD DETAILS
  - Plasmid constructs
  - Quantitative reverse transcriptase PCR (RT-qPCR)
  - Transgenic cell lines
  - Kidney organoid generation
  - Lentiviral PAX8 re-expression rescue experiments
  - Immunofluorescence analysis
  - Imaging and image analysis
  - Antibodies
  - Single cell RNA-seq sample preparation and sequencing
  - Multiome-seq sample preparation and sequencing
  - Single cell RNA-seq and multi-ome (paired single nucleus RNA-seq and single nucleus ATAC-seq) data preprocessing
  - scRNA-seq analysis
  - Integration of human fetal and organoid scRNA-seq data
  - Multi-ome (paired snRNA-seq and snATAC-seq) data analysis
  - Correlating transcription factor gene expression and corresponding motif accessibility
  - Developmental Cell *59*, 595–612, March 11, 2024 609
  - Peak to gene links and regulon analysis
  - Pseudotime trajectory analysis
- QUANTIFICATION AND STATISTICAL ANALYSIS

## Star*Methods

### Key Resources Table

**Table:** 

REAGENT or RESOURCE	SOURCE	IDENTIFIER
Antibodies		
Rabbit anti-PKC *ζ* (C-20)	Santa Cruz Biotech	Cat# sc-216; RRID:AB_2300359
Mouse anti-Integrin beta 1 [12G10]	Abcam	Cat# ab30394: RRID:AB_775726
Goat anti-TCF-2/HNF-1 beta	R&D Systems	Cat# AF3330; RRID:AB_2116774
Rabbit anti-ZO1	Invitrogen	Cat# 40-2200; RRID AB_2533456
Mouse anti-E-Cadherin	BD Bioscience	Cat# 610181; RRID:AB_397580
Goat anti-E-Cadherin	R&D Systems	Cat# AF648; RRID:AB_355504
Rabbit anti-PAX8 [EPR13511]	Abcam	Cat# ab189249; RRID:AB_2801268
Mouse anti-PAX8	Proteintech	Cat# 60145-4; RRID:AB_10643528
Mouse anti-PAX2	Developmental Studies Hybridoma Bank (DSHB)	Cat# PCRP-PAX2-1A7; RRID:AB_2722284
Mouse anti-Twist1 [Twist2C1a]	Abcam	Cat# ab50887; RRID:AB_883294
RFP Booster Alexa Fluor 568	Proteintech	Cat# rb2AF568; RRID:AB_2827576
Goat anti-GFP	Abcam	Cat# ab6673; RRID:AB_305643
Rabbit anti-GFP	Abcam	Cat# ab290; RRID:AB_303395
Rabbit anti-WT1 [CAN-R9(IHC)-56-2]	Abcam	Cat# ab89901; RRID:AB_2043201
Sheep anti-Cadherin-6/KCAD	R&D Systems	Cat# AF2715; RRID:AB_883857
Lotus Tetragonolobus Lectin (LTL), Biotinylated	Vector Laboratories	Cat# B-1325; RRID:AB_2336558
Alexa Fluor 488 donkey anti-Goat IgG (H+L)	Invitrogen	Cat# A11055
Alexa Fluor 647 donkey anti-Rabbit IgG (H+L)	Invitrogen	Cat# A32795
Alexa Fluor 488 donkey anti-Sheep IgG (H+L)	Invitrogen	Cat# A11015
Alexa Fluor 568 donkey anti-Goat IgG (H+L)	Invitrogen	Cat# A11057
Alexa Fluor 647 donkey anti-Goat IgG (H+L)	Invitrogen	Cat# A32849
Alexa Fluor 488 donkey anti-Rabbit IgG (H+L)	Invitrogen	Cat# A32790
Dylight Cy3 goat anti-Mouse IgG (H+L)	Rockland Immunochemicals	Cat# 610-104-121
Bacterial and virus strains		
One Shot TOP10 Chemically Competent E.Coli	Invitrogen	Cat# C404003
Chemicals, peptides, and recombinant proteins		
CHIR99021	Tocris	Cat# 4423
Recombinant Human FGF-9	PeproTech	Cat# AF-100-23
Geltrex	Gibco	Cat# A14133-01
DMEM F12	Gibco	Cat# 31331-028
TeSR-E6	Stemcell Technologies	Cat# 05946
Anti-Adherence Rinsing Solution	Stemcell Technologies	Cat# 07010
StemFlex Combo Kit	ThermoFisher Scientific	Cat# A3349401
TrypLE Select	Gibco	Cat# 12563029
Rock inhibitor Y27623	Calbiochem	Cat# 688000
RevitaCell	Thermo Fisher Scientific	Cat# A2644501
Lipofectamine Stem	Invitrogen	Cat# STEM00001
Opti-MEM	Gibco	Cat# 31985-062
Formaldehyde (16%)	Thermo Fisher Scientific	Cat# 28908
Doxycycline	Tocris	Cat# 4090
DMSO	Tocris	Cat# 3176
G418	Tocris	Cat# 4131
Puromycin	Tocris	Cat# 4089
Human Stem Cell Nucleofector Kit 1	Lonza	Cat# VPH-5012
Roche Rapid Ligation Kit	Roche	Cat# 11635379001
First Strand cDNA Synthesis Kit	ThermoScientific	Cat# K1612
FS Universal SYBR Green Master (Rox)	Merck Life Science	Cat# 04913850001
Super PiggyBac Transposase	System Biosciences	Cat# PB210PA-1
Deposited data		
Codes are deposited in Röper lab Github	This study	https://github.com/roeperlab/multiome-organoid and https://doi.org/10.5281/zenodo.10495881
Raw and processed data (scRNA-seq, snRNA-seq, snATAC-seq)	This study	GEO: GSE232787
Experimental models: Cell lines		
iPSC(IMR90) clone (#1)	WiCell	Cat# iPSC(IMR90)-1
PAX8-dCas9-KRAB_1 iPSCs	This study	N/A
PAX8-dCas9-KRAB _2 iPSCs	This study	N/A
PAX2-dCas9-KRAB iPSCs	This study	N/A
Oligonucleotides		
RT-qPCR primers:		
GAPDH_Fwd: AGATCATCAGCAATGCCTCCTG	This study	N/A
GAPDH_Rev: AGTCTTCTGGGTGGCAGTGA	This study	N/A
PAX8_Fwd: AGCAACCATTCAACCTCCCT	This study	N/A
PAX8_Rev: TGATCACTGTCATCCATTTTCCTCT	This study	N/A
CDH1_Fwd: GATGAAAATCTGAAAGCGGCTGA	This study	N/A
CDH1_Rev: TCTTGAAGCGATTGCCCCAT	This study	N/A
PAX2_Fwd: GCGCCGGATGTTTCTGTGA	This study	N/A
PAX2_Rev: CTGGCACTGGGGGAAAAGAA	This study	N/A
gRNA oligos:		
PAX2_F: caccGCTCTCCGACCACCGCCTCT	This study	N/A
PAX2_R: aaacAGAGGCGGTGGTCGGAGAGC	This study	N/A
PAX8_1_F: caccAGCTGGCTAGCAGTGAGGAC	This study	N/A
PAX8_1_R: aaacGTCCTCACTGCTAGCCAGCT	This study	N/A
PAX8_2_F: caccGCCGGCCAGGTATGTCACCC	This study	N/A
PAX8_2_R: aaacGGGTGACATACCTGGCCGGC	This study	N/A
Recombinant DNA		
pPB-Ins-TRE3Gp-KRAB-dCas9-ecDHFR-IRES-GFP-EF1Ap-Puro	Addgene	183410
pPB-Ins-U6p-sgRNAentry-EF1Ap-TetOn3G-IRES-Neo	Addgene	183411
pPB-Ins-U6p-sgRNA-PAX8-EF1Ap-TetOn3G-IRES-Neo_1	This study	N/A
pPB-Ins-U6p-sgRNA-PAX8-EF1Ap-TetOn3G-IRES-Neo_2	This study	N/A
pPB-Ins-U6p-sgRNA-PAX2-EF1Ap-TetOn3G-IRES-Neo	This study	N/A
Lentiviral gene expression		
pLV[Exp]- EF1a>hPAX8[NM_003466.4](ns):T2A:mCherry:oPRE	This study (Vectorbuilder)	VB230703-1153nzf
pLV[Exp]-EF1a>mCherry:oPRE	This study (Vectorbuilder)	VB230703-1156har
Software and algorithms		
Imaris (v10.0.1)	Bitplane	https://imaris.oxinst.com/
Fiji (v2.00-rc-69/1.52p)	Image J	https://imagej.net/software/fiji/
Prism (v9.5.0)	Graphpad Software	https://www.graphpad.com
R (v4.2.1)	The R Foundation	https://www.r-project.org/
gplot2 (v3.4.0)	Wickham^68^	https://cran.r-project.org/web/packages/ggplot2/index.html
G:Profiler (ve108_eg44_p17_9f356ae)	Randvere et al.^69^	https://biit.cs.ut.ee/gprofiler/gost
ArchR (v1.0.3)	Granja et al.^24^	https://www.archrproject.com/
TFBSTools (vl.36.0)	Tan and Lenhard^70^	https://rdrr.io/bioc/TFBSTools/
ChromVAR (v1.20.2)	Schep et al.^71^	https://greenleaflab.github.io/chromVAR/
HOMER (V4.11.1)	Heinz et al.^72^	http://homer.ucsd.edu/homer/motif/
pheatmap (v1.0.12)	Raivo Kolde	https://github.com/raivokolde/pheatmap
Seurat (v4.3.0)	Stuart et al.^73^	https://satijalab.org/seurat/index.html
Cell Ranger (v7.0.1)	10X Genomics	https://support.10xgenomics.com/single-cell- https://support.10xgenomics.com/single-cell-gene-expression/software/pipelines/latest/installation
Cell Ranger ARC (v2.0.2)	10X Genomics	https://support.10xgenomics.com/single-cell-multiome-atac-gex/software/pipelines/latest/installation
Other		
Cell culture plastics	N/A	N/A
AggreWell 400 6-well plate	Stemcell Technologies	Cat# 34421
AggreWell 400 24-well plate	Stemcell Technologies	Cat# 34411
6 well ultra-low-adhesion plate	Greiner Bio-One	Cat# 657185
Counting slides	Invitrogen	Cat# 10228
Countess II	Life Technologies	AMQAX1000
EVOS FL	Life Technologies	AMF4300R
QIAprep Spin Miniprep Kit	Qiagen	27104
QIAprep Plasmid Plus Midiprep Kit	Qiagen	12943
RNeasy Mini Kit	Qiagen	74104
RNase-Free DNase Set	Qiagen	79254

## Resource Availability

### Lead contact

Further information and requests for resources and reagents should be directed to and will be fulfilled by the lead contact, Katja Röper, kroeper@mrc-lmb.cam.ac.uk.

### Materials availability

All unique/stable reagents generated in this study are available from the lead contact with a completed materials transfer agreement.

### Data and code availability

Single-cell/single nucleus and ATAC RNA-seq data have been deposited at GEO and are publicly available as of the date of publication. Accession numbers are listed in the key resources table. Microscopy data reported in this paper will be shared by the lead contact upon request.

All original code has been deposited at github and is publicly available as of the date of publication. The DOI is listed in the key resources table.

Any additional information required to reanalyze the data reported in this work paper is available from the lead contact upon request.

## Experimental Method and Study Participant Details

### Cell lines

iPS(IMR90)-1 (IMR-90-1) cells were obtained from WiCell, and were maintained in StemFlex (ThermoFisher, A3349401) containing 1x Antibiotic-Antimycotic (Anti-/Anti-; Gibco, 15240062) on Matrigel- or Geltrex-coated plates. Cells were passaged every 3-4 days using EDTA and re-seeded in StemFlex containing 1x Anti-/Anti- and 10μM ROCK inhibitor Y27623 (ROCKi; Calbiochem, 688000). PAX8-dCas9-KRAB-puro (PAX8-dCas9-KRAB_1 and PAX8-dCas9-KRAB_2) iPSCs and PAX2-dCas9-KRAB-puro (PAX2-dCas9-KRAB) iPSCs, generated as described below, were maintained in StemFlex containing 1x Anti-/Anti, 0.5 μg/mL puromycin (Tocris, 4089) and 200 μg/mL G418 (Tocris, 4131). Cells were passaged every 3-4 days using EDTA and re-seeded in StemFlex containing 1x Anti-/Anti-, 10 μM ROCKi, 0.5 μg/mL puromycin (Tocris, 4089) and 200 μg/mL G418 (Tocris, 4131). All cell cultures were incubated at 37°C with 5% CO_2_.

## Method Details

### Plasmid constructs

pPB-Ins-U6p-sgRNAentry-EF1Ap-TetOn3G-IRES-Neo was a gift from Azim Surani (Addgene plasmid # 183411; http://n2t.net/addgene:183411; RRID:Addgene_183411). pPB-Ins-TRE3Gp-KRAB-dCas9-ecDHFR-IRES-GFP-EF1Ap-Puro was a gift from Azim Surani (Addgene plasmid # 183410; http://n2t.net/addgene:183410; RRID:Addgene_183410). All oligos used for cloning were ordered using the custom DNA oligo synthesis service from Merck and are listed in the “Oligonucleotides” section of the key resources table. Specific gRNA sequences for dCas9-KRAB mediated gene repression were designed by identifying putative transcription start sites (TSSs) for target genes according to FANTOM CAGE data publicly available on ZENBU,^74^ then the Wellcome Sanger Institute Genome Editing (WGE) CRISPR Finder^75^ was used to identify CRISPR sequences 50-100bp downstream of a putative TSS for optimal gene knockdown as previously described.^76^ At least 2 gRNA sequences were tested per TSS. For generation of gRNA expression vectors, complementary gRNA oligos were annealed and ligated into pPB-Ins-U6p-sgRNAentry-EF1Ap-TetOn3G-IRES-Neo (Addgene plasmid, 183411) by Esp3I digestion (NEB, R0734) followed by ligation with Rapid DNA Ligation Kit (Roche, 11635379001). TOP10 chemically competent E. coli (Thermo Fisher, C404010) were used for transformation. Successful transformants were purified using QIAprep Spin Miniprep Kit (Qiagen, 27104) or QIAprep Plasmid Plus Midiprep Kit (Qiagen, 12943), then validated with Sanger sequencing.

### Quantitative reverse transcriptase PCR (RT-qPCR)

To isolate RNA for RT-qPCR analysis, cells/organoids were washed twice with PBS and then lysed in 350 μl RLT lysis buffer prior to RNA isolation with RNeasy Mini Kit (Qiagen, 74104) with on-column Dnase digestion using the Rnase-free Dnase set (Qiagen, 79254). Complementary DNA (cDNA) was generated using First Strand cDNA Synthesis Kit (Thermo Scientific, K1612) with 1 μg RNA for each sample. RT-qPCR was performed using SYBR Green (Roche, FSUSGMMRO) with forward and reverse primers diluted to final concentration of 1μM per reaction, with 5.2 μl diluted cDNA, in MicroAmp Optical 384-well Reaction Plate with Barcode (Applied Biosystems, 43309849) with MicroAmp Optical Adhesive Film (Applied Biosystems, 4311971) overlaid. Reactions were performed in an Applied Biosystems ViiA7 machine with the following cycling conditions: Hold for 10 minutes at 95°C, then 10 seconds at 95°C and 20 seconds at 58°C repeated for 40 cycles, followed by a melt curve stage. The DDCt method^77^ was used to analyse PCR data using *GAPDH* as a housekeeping gene. RT-qPCR data were analysed using Prism (Graphpad, v9.5.0).

### Transgenic cell lines

For establishment of PAX8-dCas9-KRAB_1, PAX8-dCas9-KRAB_2 and PAX2-dCas9-KRAB lines, 5x10^5^ IMR-90-1 cells were seeded into Matrigel- or Geltrex-coated 6-well plates in StemFlex with ROCKi, and the following day were washed with PBS and incubated in E8 media (Gibco, A1517001) for at least 1 hour. For each well of a 6-well plate, cells were then transfected with the plasmids Super piggyBac Transposase expression vector (Stratech, PB210PA-1-SBI) (500ng), pPB-Ins-U6p-sgRNA-PAX8-EF1Ap-TetOn3G-IRES-Neo_1, pPB-Ins-U6p-sgRNA-PAX8-EF1Ap-TetOn3G-IRES-Neo_2, or pPB-Ins-U6p-sgRNA-PAX2-EF1Ap-TetOn3G-IRES-Neo gRNA expression plasmids (1μg) and pPB-Ins-TRE3Gp-KRAB-dCas9-ecDHFR-IRES-GFP-EF1Ap-Puro (1.5μg) mixed with 10μl Lipofectamine Stem transfection reagent (Life Technologies, STEM00003) in 500μl Opti-MEM with 1x Revitacell supplement for 20 minutes followed by addition of StemFlex with 1x Revitacell supplement. The following day, media were replaced with fresh StemFlex with 1x Revitacell supplement. 48 hours later, media were refreshed with fresh StemFlex with 1x Revitacell supplement and containing antibiotics 0.5 μg/mL puromycin and 200 mg/mL G418 for selection. Media were refreshed with selection media every 2-3 days thereafter. Around a week later, surviving colonies for each well were dissociated together (effectively pooling multiple colonies) and re-seeded into 2 wells. Once cells reached ~80% confluence, knockdown validation. For knockdown validation, transgenic lines were seeded for organoid generation into 3 wells, differentiated to Day 7 with 1 well additionally treated with 1μM Dox, and another well treated with 4μM Dox, then all harvested for RNA extraction. For knockdown validation, RT-qPCR was performed with primers specific to the gene of interest according to the “Quantitative reverse transcriptase PCR (qRT-PCR)” section below. PAX8-dCas9-KRAB_1 and PAX8-dCas9-KRAB_2 iPSCs showed similar knockdown efficiency and knockdown phentoypes, so only data from PAX8-dCas9-KRAB_1 are presented in this paper.

Piggybac transposase facilitates the integration of a transposon-containing donor cassette specifically at ’TTAA’ sites which are randomly dispersed in the genome, and therefore the activity of the Dox-inducible dCas9 cassette may be subject to genomic position effects leading to heterogenous responsiveness to Dox and consequently variable knockdown efficiency. To improve knockdown efficiency of PAX8-dCas9-KRAB_1 and PAX2-dCas9-KRAB iPSCs, cells were incubated with 1μM Dox for 48 hours to induce the expression of the dCas9-KRAB cassette which includes cytoplasmic GFP as a readout for Dox responsiveness. Cells were then dissociated to single cells, and the top 20% of GFP^+^ cells isolated by flow cytometry, followed by culture on Matrigel- or Geltrex-coated plates in StemFlex containing 1x Anti-/Anti- and 1x Revitacell (Gibco, A2644501). Top 20% sorted PAX2-dCas9-KRAB cells were used to generate all PAX2-dCas9-KRAB knockdown data presented in this paper (Figures S5D and S5E). Top 20% sorted PAX8-dCas9-KRAB_1 cells differentiated to organoids showed near complete knockdown of *PAX8* and completely lacked epithelial structures.

### Kidney organoid generation

Kidney organoids were generated using a previously published protocol with modifications.^5^ Human iPSCs were grown to ~70% confluency and dissociated with EDTA (Day -1), counted using Countess II (Life Technologies, AMQAX1000), then seeded evenly onto Geltrex-coated 6-well plates at 100,000 cells per cm^2^ in 2ml StemFlex with ROCKi. The following day (Day 0), cells were washed with PBS, then incubated in 2ml fresh organoid induction media A: TeSR-E6 (Stemcell Technologies, 05946) with 1x Anti-/Anti- and 6μM CHIR99021 (Tocris, 4423). Media were refreshed on Day 3. On Day 4, media were replaced with organoid induction media B: TeSR-E6 with 1x Anti-/Anti-, 1μM CHIR and 200ng/ml FGF9 (PeproTech, AF-100-23). Media were refreshed on Day 6. On Day 7, cells were washed with PBS and dissociated with TrypLE (3 mins at 37°C), spun down and resuspended in organoid induction media B with 10μM ROCKi, and counted. Cells were then seeded into Aggrewell-400 24-well plates (Stemcell Technologies, 34411) or 6-well plates (Stemcell Technologies, 34421) that were pre-coated with Anti-adherence solution (Stemcell Technologies, 07010), at 1.2x10^6^ cells per well (24-well) or 7x10^6^ per well (6-well). Plates were centrifuged at 200g for 5 mins, then incubated at 37°C with 5% CO_2_. At Day 10, the resulting organoids were gently washed out of Aggrewell plates, washed once with PBS, and transferred at ~3000 organoids per well into 6-well ultra-low-adhesion plates (Greiner Bio-One, 657185) in fresh organoid induction media B, and incubated on a shaker at 100rpm, at 37°C with 5% CO_2_. At Day 12, media were replaced with TeSR-E6 with 1x Anti-/Anti-. Media were refreshed with TeSR-E6 with 1x Anti-/Anti- at Day 14, and every 2-3 days thereafter. This protocol first generates intermediate mesoderm as indicated by the induction of *OSR1* gene expression (peaking at day 3), followed by generation of nephron progenitor cells, indicated by expression of *SALL1* (peaking at day 5) and subsequent induction of its downstream target *SIX2* (rising from day 6; Figure S1A), which are expressed in both uncommitted and committed nephron progenitor cells *in vivo*.^20^

### Lentiviral PAX8 re-expression rescue experiments

To test specificity of the PAX8-dCas9-KRAB knockdown approach, PAX8-dCas9-KRAB_1 iPSCs were differentiated in the kidney organoid protocol, with 1μM Dox added at Day 4 to induce the knockdown cassette. At Day 7, cells were transferred to Aggre-well-400 24-well plates, and custom-designed lentiviruses (constructed and packaged by Vectorbuilder, Chicago, USA) were added that contained a PAX8 overexpression cassette with cytoplasmic mCherry as a readout for successful transduction driven by the human EF1α promoter (EF1α::PAX8-T2A-mCherry [pLV[Exp]- EF1a>hPAX8[NM_003466.4](ns):T2A:mCherry:oPRE]), at an MOI of 11.5. Lentivirus constructs containing cytoplasmic mCherry only driven by the human EF1α promoter (EF1α::mCherry [pLV[Exp]-EF1a>mCherry:oPRE]) were used as a negative control; in pilot experiments transduction efficiency was found to be substantially higher for the control lentivirus and so an MOI of 2.4 was used. Resulting organoids were transferred to suspension at Day 10 and cultured to Day 14 as per the normal protocol with the addition of 1μM Dox, and then all organoids were harvested at Day 14 for immunofluorescence analysis (see below).

### Immunofluorescence analysis

To harvest for immunofluorescence (IF) analysis, organoids were washed once with PBS, fixed in 3% PFA overnight at 4°C, then washed in PBS (3x10 min) and stored in PBS with 0.01% sodium azide. Prior to antibody staining, samples were incubated in blocking buffer overnight at 4°C (PBS containing 0.25% Triton-X and 5% BSA). Samples were incubated in primary antibodies diluted in wash buffer (PBS containing 0.1% Triton-X and 5% BSA) at the indicated concentrations overnight at 4°C. Next, samples were washed in wash buffer (3x15 min at room temperature) and incubated in secondary antibodies diluted in wash buffer at the indicated concentrations, with DAPI, overnight at 4°C. Then, samples were washed in wash buffer (3x15 min at room temperature) and mounted on glass slides in Vectorshield (Vector Labs, H1000) in 120μm spacers (Invitrogen, S24735) with coverslips overlaid.

### Imaging and image analysis

Brightfield images were taken using an EVOS FL microscope (Life Technologies, AMF4300R). Confocal images were taken using a Zeiss LSM 710 or 780, or a Nikon W1 Spinning Disk microscope. Image files were analysed using Fiji (Image J) or Imaris (Bitplane, Oxford Instruments, v10.0.1). To quantify E-Cadherin-positive volume, confocal z-stacks of individual organoids were first obtained using a Nikon W1 Spinning Disk microscope with imaging at 1 μm intervals, the image files were then imported into Imaris, and the surfaces option was used to volume render and quantify the E-Cadherin signal using default settings where possible. For rescue experiments, to quantify the proportion of E-Cadherin-positive volume that was also GFP^+^, the E-Cadherin^+^ and GFP^+^ volumes were volume rendered separately, using default settings where possible, and then the overlap volume was quantified and expressed as a proportion of total E-Cadherin-positive volume per organoid. All images presented are representative of at least 2 independent organoid batches. Unless otherwise indicated, immunofluorescence images shown are representative single confocal sections.

### Antibodies

Primary antibodies used for protein detection, with their corresponding dilutions for immunofluorescence (IF) were as follows: rabbit anti-PKC ζ (C-20) (Santa Cruz Biotech, sc-216, 1:200), mouse anti-Integrin beta 1 [12G10] (Abcam, ab30394, 1:200), goat anti-TCF-2/HNF-1 beta (R&D Systems, AF3330, 1:200), rabbit anti-ZO1 (Invitrogen, 40-2200, 1:200), mouse anti-E-Cadherin (BD Bioscience, 610181, 1:200), goat anti-E-Cadherin (R&D Systems, AF648, 1:200), rabbit anti-PAX8 [EPR13511] (Abcam, ab189249, 1:200), mouse anti-PAX8 (Proteintech, 60145-4-Ig, 1:200), mouse anti-PAX2 (Developmental Studies Hybridoma Bank (DSHB), PCRP-PAX2-1A7, 1:20), rabbit anti-GFP (Abcam, ab290, 1:200), rabbit anti-WT1 [CAN-R9(IHC)-56-2] (Abcam, ab89901, 1:200), sheep anti-Cadherin-6/KCAD (R&D Systems, AF2715, 1:200). Biotinylated Lotus Tetragonolobus Lectin (LTL; Vector Laboratories, B-1325-2, 1:200) was used to stain proximal tube cells. Alexafluor 488, 568 and 647 (Invitrogen), and Dylight Cy3 (Rockland Immunochemicals) secondary antibodies were used for detection of primary antibodies.

### Single cell RNA-seq sample preparation and sequencing

Organoids from batch 1 (days 10, 14, 24) and from batches 2 and 3 (day 24) were collected for single cell RNA-seq. For each sample, ~5000 organoids were collected in 15ml tubes, washed once with PBS then dissociated to single cells in 500μL Bacillus Licheniformis protease (Sigma, P5380) on ice for 20 min,^78^ with gentle agitation by pipetting every 2 min, followed by resuspension in PBS with 10% FBS to neutralise the protease. Cells were centrifuged at 300g for 5 min, then resuspended in PBS with 10% FBS and filtered through a 30μm strainer prior to centrifugation and final resuspension in PBS with 0.5% BSA. Cells were counted with Countess II (Life Technologies) to ensure cell viability > 90% and to achieve a targeted cell recovery of 1,600 cells per sample for batches 1 and 2, and 5,000 cells per sample for batch 3. Cells were processed for library preparation using a 10X Chromium sc 3’mRNA kit (v3, 10X Genomics) at the Cancer Research UK Genomics Facility, Cambridge, UK.

### Multiome-seq sample preparation and sequencing

Organoids from batch 2 (days 10, 12, and 14) and batch 3 (days 10, 12, 14 DMSO, and 14 CHIR), were collected for single nucleus Multiome-seq. Single cell preparations were generated as above, then further processed for single nucleus isolation by centrifugation at 300g for 4 min at 12°C and resuspension in 100μL freshly prepared ice-cold lysis buffer (10mM Tris-HCL, 10mM NaCl, 3mM MgCl2, 0.1% Tween-20, 1 % BSA, 1mM DTT, 1 U/μL Protector RNase Inhibitor (Sigma, 3335402001), 0.1% Nonidet P40 Substitute (Sigma, 74385) and 0.01% Digitonin in nuclease-free H_2_O) followed by incubation on ice for 5 min. Lysis was halted by addition of 900μL chilled wash buffer (10mM Tris-HCL, 10mM NaCl, 3mM MgCl2, 0.1% Tween-20, 1 % BSA, 1mM DTT, 1 U/μL Protector RNase Inhibitor in H_2_O), followed by 3 rounds of centrifugation at 500g for 5 min at 4°C and resuspension in chilled wash buffer, with the final resuspension in 50μL chilled 1x Nuclei Buffer (10X Genomics, 2000153) with 1mM DTT and 1 U/μL Protector RNase Inhibitor in H_2_O, according to the 10x Genomics demonstrated protocol for Nuclei Isolation for Single Cell Multiome (ATAC + GEX) sequencing (CG000365, RevA). After resuspension in 1x Nuclei Buffer, nuclei were stained with Trypan Blue and counted using a Countess II (Life Technologies) to confirm complete lysis and to achieve a targeted nuclei recovery of 1,600 nuclei per sample for batch 2, and 5,000 nuclei per sample for batch 3. Nuclei were processed for library preparation using a 10X Chromium scMultiome kit (10X Genomics) at the Cancer Research UK Genomics Facility.

All samples from a given batch were sequenced together (RNA and ATAC libraries were sequenced separately) on an Illumina NovaSeq 6000 to an aimed depth of 100,000 – 150,000 reads per cell.

### Single cell RNA-seq and multi-ome (paired single nucleus RNA-seq and single nucleus ATAC-seq) data preprocessing

De-multiplexed scRNA-seq FASTQ files were run in the Cell Ranger pipeline (10X Genomics, v7.0.1) to produce barcoded count matrices of RNA data. Paired de-multiplexed snRNA- and snATAC-seq FASTQ files were run in the Cell Ranger ARC pipeline (10X Genomics, v2.0.2) to produce barcoded count matrices of RNA data and fragment files of ATAC data. All reads were aligned to GRCh38/hg38.

### scRNA-seq analysis

For analysis of organoid transcriptomes at days 10, 14 and 24 combined across 3 batches (Figures S1C–S1N), filtered feature-bar-code matrices of scRNA-seq data (batch 1, days 10, 14, and 24; batches 2 and 3, day 24) and snRNA-seq data (batches 2 and 3, days 10 and 14) were converted into individual Seurat objects using the Seurat R package (version 4.3.0)^73^ and quality control filtered for cells containing > 1000 genes (> 2500 genes for batch 1, day 14) and < 15% mitochondrial reads. All samples were then merged into a single Seurat object, then split using the SplitObject function according to batch. Gene expression counts were normalized for each batch separately using the NormalizeData function. Features that were repeatedly variable across batches were identified using the SelectIntegrationFeatures function, which were then used to scale each batch separately using the ScaleData function and to run PCA. Samples within each batch were integrated using reciprocal PCA; the 3 integrated batches were then integrated using canonical correlation analysis. Graph-based clustering was performed on the final integrated object using the top 44 principal components at a resolution of 0.02 to generate 2 clusters. Marker genes for each cluster were identified using the FindMarkers function with a minimum fraction of 0.2 and a minimum difference fraction of 0.1, which were used to annotate the clusters as either epithelial or stromal lineages. The epithelial lineage cluster was then subset and re-clustered using the top 28 principal components at a resolution 0.2. Marker genes for each cluster were identified using the “FindAllMarkers” function with a minimum fraction of 0.5 and a log_2_ fold change of 1. Cluster identities were then manually annotated by querying resulting marker genes using the Human Nephrogenesis Atlas (https://sckidney.flatironinstitute.org/)^28^ and by assessing cell cycle phase scored using the “CellCycleScoring” function in Seurat (Figure S1K)

### Integration of human fetal and organoid scRNA-seq data

Reference data from Stewart et al.^21^ was pre-processed using the scanpy framework, and pseudotime calculated using scFates.^79^ Using reference PCA embeddings, organoid scRNAseq data was asymmetrically integrated using the “sc.tl.ingest” function. Label prediction between scFates milestones in fetal nephron data and scRNAseq profiles from organoids was performed using a multilayer perceptron in sklearn as previously described.^80^ Pseudotime trajectories were aligned between fetal data and organoid data by first calculating genes that vary significantly over fetal nephron pseudotime with switchDE,^81^ taking genes with a q value < 0.05. Trajectories were aligned using cellAlign,^82^ using 500 interpolated nodes.

### Multi-ome (paired snRNA-seq and snATAC-seq) data analysis

Paired snRNA-seq and snATAC-seq datasets (batches 2 and 3, days 10, 12, and 14) were analyzed using ArchR (version 1.0.3)^24,83^ with default settings for all functions used unless otherwise stated. The RNA count matrices and ATAC fragment files output from Cell Ranger were used to generate Arrow files for each sample. Initially, all Arrow files (batches 2 and 3, all time points) were combined into one ArchR project, and batch correction with Harmony^84^ as implemented in ArchR was applied but was found to be inadequate to resolve batch effects without losing biological information. Batches were therefore analysed independently, which revealed similar results for each batch, so data from batch 3 are shown unless otherwise indicated. For each batch separately, Arrow files for days 10, 12 and 14 were combined into an ArchR project and quality control filtered for nuclei with 1,000-10,000 genes, >5,000 RNA transcripts, TSS enrichment >6, and >2,500 ATAC fragments. Dimensionality reduction was performed with the “addIterativeLSI” method for ATAC and RNA data separately at a resolution of 0.2, using 2500 variable features for RNA data and 25000 variable features for ATAC data. For batch 3 only, Harmony^84^ was used to remove batch effects between samples but preserve the structure of the data with regards to time point (groupBy = “Sample”, theta = 120, lambda = 0.3). For each batch, UMAP dimensionality reduction was then performed (minDist = 0.8, dims = c(1:30)). Clustering was performed at a resolution of 0.9, and poor quality and stressed cell clusters were identified and removed from the dataset. The remaining cells were again subjected to UMAP dimensionality reduction (minDist = 0.8, dims = c(1:28)) followed by clustering at a resolution of 0.1 to generate 6 clusters corresponding to stromal and epithelial lineages each at day 10, 12 and 14 for batch 3; as we were focussing on the epithelial lineage, all stromal clusters were merged into 1 cluster for subsequent analysis (Figure 1E). Analogous processing produced 5 clusters in batch 2 data which were used for downstream analysis including pseudotime trajectory inference (Figures S4B and S6A–S6C). Gene expression values for visualization were imputed using MAGIC^85^ and embedding plots were generated with the “plotEmbedding” function (quantCut = c(0.05,0.95)). Gene ontology analysis was performed by analysing gene lists in g:Profiler (https://biit.cs.ut.ee/gprofiler/gost).^69^

ATAC data were used to generate pseudobulk replicates with the “addGroupCoverages” and “addReproduciblePeakSet” functions. Chromatin accessibility peaks on chromosomes 1-22 and X and outside of blacklist regions were called with MACS2.^86^ To calculate enrichment of chromatin accessibility of transcription factor motifs on a per cell basis, ChromVAR^71^ was run with “addDeviationsMatrix”, using motif sets from JASPAR 2020 core and unvalidated collections (as currently supported within ArchR)^87^ together with PAX2, MAFB, WT1 and TWIST2 motifs, which were not present in the JASPAR 2020 human collection and so were manually generated using position frequency matrices of mouse motifs from JASPAR 2020 and then converted into position weight matrices using PWMatrixList from the TFBSTools package (v1.36.0).^70^ The SALL1 motif was taken from a previously published mouse Sall1 motif sequence.^88^ To visualize motif deviations, scores were imputed using MAGIC, and the “plotEmbedding” function was used (quantCut = c(0.05,0.95)). Marker genes for clusters as indicated in the text were identified using the “getMarkerFeatures” function using the Wilcoxon test and correcting for TSS Enrichment, and ATAC and RNA sequencing depth and filtered using the “getMarkers” function (cutOff =“FDR <= 0.01 & Log_2_FC >= 1”). Marker peaks of clusters as indicated in the text were identified using the “getMarkerFeatures” function using the Wilcoxon test and correcting for TSS Enrichment, and ATAC and RNA sequencing depth, followed by filtering with the “getMarkers” function (cutOff = “FDR <= 0.01 & Log_2_FC >= 1”). Enrichment of transcription factor motifs within marker peaks for each cluster was determined using the hypergeometric test using the “peakAnnoEnrichment” function based on position frequency matrices from the JASPAR 2020 motif set, with all peaks used as a background.

Vierstra transcription factor archetypes^7^ were added using “addMotifAnnotations” (motifSet = “vierstra”). Once added, enrichment of Vierstra archetypes was determined using the “getMarkerFeatures” function using the Wilcoxon test and correcting for TSS Enrichment, and ATAC and RNA sequencing depth, followed by filtering with the “getMarkers” function (cutOff = “FDR <= 0.01 & Log_2_FC >= 1”). Transcription factor footprinting was performed and visualized using the ArchR functions “getFootprints” and “plotFootprints”, with the Tn5 insertion signal subtracted from footprinting signals prior to plotting to correct for Tn5 insertion bias (normMethod = “subtract”).

### Correlating transcription factor gene expression and corresponding motif accessibility

To identify activator and repressor transcription factors, correlations between transcription factor gene expression and chromatin accessibility of corresponding motifs were derived using the “correlateMatrices” function. Resulting transcription factors were filtered according to a correlation adjusted p value of 0.001, and further filtered using the “getMarkers” function by correlation value (>0.2 or < -0.2 to classify transcription factors as activators or repressors, respectively) and Log2fold change in the epithelial lineage compared to the stromal lineage (>1.5 or <-1.5 for epithelial enriched or stromal enriched transcription factors, respectively) (Figure 2D).

### Peak to gene links and regulon analysis

Peak to gene links were defined as chromatin accessibility peaks within 500kb upstream or downstream of a gene TSS with accessibility that correlates with expression of that gene, and were identified using the “addPeak2GeneLinks” function (maxDist = 500000) and filtered according to false discovery rate <1x10^-4^ and correlation value >0.4 (Figure S5F). Filtered peak to gene links were then analysed for overrepresentation of specific transcription factor motifs within HOMER (v4.11.1)^72^ using the “findMotifsGenome.pl” function with HOMER Motif files as input. Sequencing tracks of chromatin accessibility were generated in ArchR using the “plot-BrowserTrack” function and were normalized by the total number of reads in TSS regions.

### Pseudotime trajectory analysis

To analyse dynamics of transcription factor activity along the renal mesenchymal-to-epithelial transition, pseudotime analysis was performed using ArchR. For batch 3 (Figure 5), the ArchR object was clustered at a resolution of 0.3, and the “addTrajectory” function applied using the following user-defined trajectory as a guide for the supervised trajectory analysis: “Cluster 3”, “Cluster 4”, “Cluster 1”. For batch 2 (Figure S6), the ArchR object was clustered at a resolution of 0.2 and the “addTrajectory” function used with the following supervised trajectory: “Cluster 3”, “Cluster 4”, “Cluster 5”. Transcription factor gene expression and corresponding motif accessibility (ChromVAR deviation scores) were correlated over pseudotime using “getTrajectory” and “correlateTrajectories” using “varCutOff = 0.55” for both RNA and motif matrices. Transcription factors were filtered according to correlation false discovery rate < 0.001, and fold enrichment of motif accessibility in epithelial over stromal lineage as determined by the “peakAnnoEnrichment” function >1. Transcription factors were further filtered according to correlation value, with a value of >0.2 used to identify activators, and <-0.2 to identify repressors. Activators or repressors were ordered according to gene expression along pseudotime, and the R package pheatmap (v1.0.12) was used to generate paired heatmaps of gene expression and corresponding motif accessibility along pseudotime (Figure 5). Gene expression and motif accessibility (as ChromVar deviations) were visualised along pseudotime using the “plotTrajectory” function.

## Quantification and Statistical Analysis

Statistical analyses of RNA-seq and ATAC-seq data were performed within ArchR. Marker genes or peaks as presented within heatmaps were analysed using two-sided Wilcoxon rank-sum test, with thresholds for heatmaps set at log_2_ fold change > 1 and false discovery rate < 0.01. In violin plots of gene expression determined by snRNA-seq, box centre lines represent median, limits represent upper and lower quartiles, and whiskers represent 1.5x interquartile range. Statistical analyses on image quantifications, and RT-qPCR data, were performed within Prism. Data were assumed to have normal distribution, and so where two groups are compared, data were analysed using unpaired t-test. In plots of image quantifications, dots represent data from individual organoids, with bars representing mean ± SEM. In plots of RT-qPCR data, dots represent data from each batch normalised to corresponding control, with bars representing mean ± SEM. In figure legends, “n” is used to represent the number of individual organoids, the number of independent batches is also indicated. Asterisks in plots represent p values as follows: * (p < 0.05), ** (p < 0.01), *** (p < 0.001), **** (p < 0.0001). All the details of quantifications and statistical analyses are fully described in the main text, figure legends, and method details section.

## Supplementary Material

Supplemental information can be found online at https://doi.org/10.1016/j.devcel.2024.01.011.

Document S1. Figures S1-S8

Table S1

Table S2

Table S3

Table S4

Table S5

Table S6

## Highlights

- Human iPSC-derived kidney organoids faithfully recapitulate renal MET
- Dynamic activity of transcriptional activators and repressors controls progression of MET
- PAX8 initiates renal MET downstream of Wnt/β-catenin signaling
- Wnt signaling must be downregulated for proper completion of MET via HNF1B and TEAD

## In brief

How the genome encodes human nephron structure and function is incompletely understood. Using multiomic analysis of human renal organoids and fetal kidneys, Ng-Blichfeldt et al. map transcriptional dynamics controlling the emergence of nephron epithelia from mesenchymal progenitors during human kidney development, revealing critical roles for PAX8 and transient Wnt signaling.
