# Supplementary material for: Identification of a core transcriptional program driving the human renal mesenchymal-to-epithelial transition
Source: Dev Cell. Author manuscript; Available in PMC 2024 May 29. (PMC7616043; doi:10.1016/j.devcel.2024.01.011)
Supplement: Document S1. Figures S1-S8 [file EMS196335-supplement-Document_S1__Figures_S1_S8.pdf]

Developmental Cell, Volume *59*

Supplemental information

Identification of a core transcriptional program driving the human renal mesenchymal-to-epithelial transition

John-Poul Ng-Blichfeldt, Benjamin J. Stewart, Menna R. Clatworthy, Julie M. Williams, and Katja Röper

Supplemental Figure Titles and Legends

Supplemental Figure S1, related to Figure 1. Single cell RNA-seq characterisation of renal organoids.

(A) Time course RT-qPCR data for the intermediate mesoderm marker *OSR1,*and the nephron progenitor markers *SALL1* and *SIX2* over the period of the kidney organoid protocol. Dots and bars represent mean ± SEM, N = 2-4 independent organoid batches at each time point.

(B) Representative immunofluorescence image of a renal organoid at day 24 stained for WT1 (green), LTL (white), CDH1 (red) and DAPI (blue). Scale bar is 30µm.

(C) UMAP plot of 23,856 scRNA-seq and snRNA-seq cells harvested across 3 organoid batches, harvested at days 10, 14 and 24, coloured by batch.

(D) Violin plots showing number of genes per batch.

(E) Violin plots showing percentage mitochondrial reads per batch.

Box centre line, median; limits, upper and lower quartiles; whiskers, 1.5x interquartile range

(F) UMAP plot of 23,856 scRNA-seq and snRNA-seq cells as in (C), analysed by coarse clustering with resulting clusters annotated as epithelial or stromal lineage.

(G) Heatmap showing top 10 genes that define the epithelial and stromal lineages.

(H) UMAP plots of 23,856 scRNA-seq and snRNA-seq cells as in (C), coloured by *PAX8* expression (top) and *TWIST1* expression (bottom).

(I) UMAP plot of 15,917 scRNA-seq and snRNA-seq cells corresponding to the epithelial lineage as in (F), re-projected and coloured according to time point.

(J) UMAP plots of 15,917 6 scRNA-seq and snRNA-seq cells as in (I), coloured by *MAFB* expression (top left), *HNF4A* expression (top right), *TFAP2B* expression (bottom left) and *GATA3* expression (bottom right).

(K) UMAP plots of 15,917 6 scRNA-seq and snRNA-seq cells as in (I), coloured by cell cycle phase.

(L) UMAP plots of 15,917 6 scRNA-seq and snRNA-seq cells as in (I), coloured by unsupervised clustering, annotated according to querying marker genes for each cluster with the Human Nephrogenesis Atlas (https://sckidney.flatironinstitute.org) (Lindstrom et al., 2021) and assessment of cell cycle phase.

(M) Dot plot of gene expression for epithelial genes in 15,917 6 scRNA-seq and snRNA-seq cells as in (D) separated by time point.

(N) Dot plot of gene expression for nephron marker genes in 15,917 6 scRNA-seq and snRNA-seq cells as in (D) separated by time point.

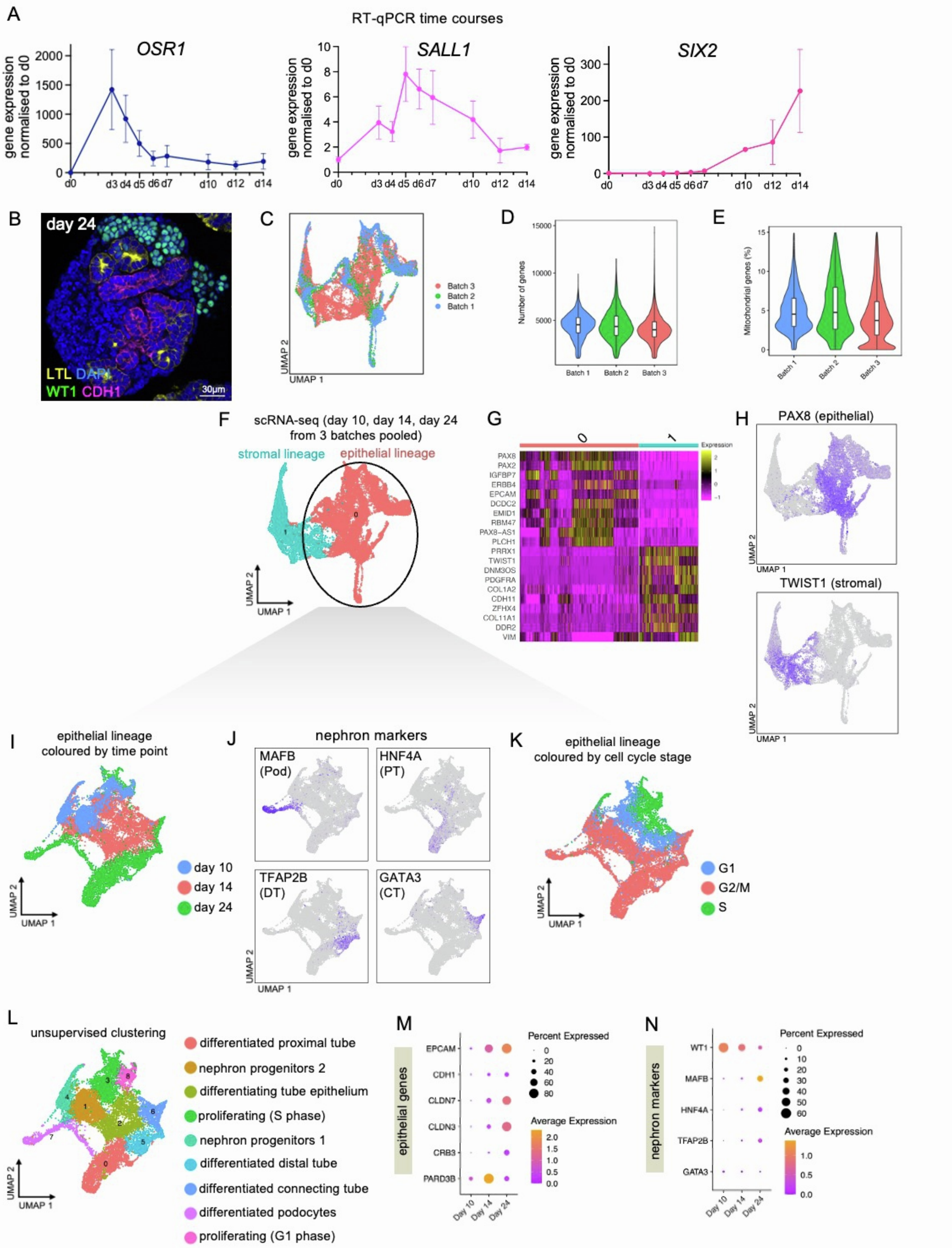

Ng-Blichfeldt et al_Supplemental Figure S1

Supplemental Figure S2, related to Figures 1 and 3. Comparison of single cell RNA-seq data of human renal organoids to human fetal kidney *in vivo.*

(A) UMAP plot of 17,994 scRNA-seq cells from 6 human fetal kidney samples collected between post conception week (pcw) 7 and 16, split by epithelial and stromal lineages, coloured by cell type annotations (Stewart et al., 2019).

(B) UMAP plot of 17,994 scRNA-seq cells as in (A), coloured by gene expression of the stromal marker *TWIST1* (left) or the nephron marker *PAX8* (right).

(C, C’) Asymmetric integration of kidney organoid scRNA-seq data split by time point with human fetal kidney scRNA-seq data, coloured by point density of organoid data (C). Cell clusters are labelled in (C) and the relevant ones shown again annotated onto the UMAP from (A) in panel (C’).

(D) UMAP plots of 8,862 nephron epithelial cells from (A), isolated and re-projected, and coloured by gene expression of nephron marker genes (related to Fig. 3). (D’) shows again the cell type legend for the clusters as also shown in Fig. 3A, and (D’’) shows an enlarged comparison of the *PAX2* and *PAX8* expression profiles.

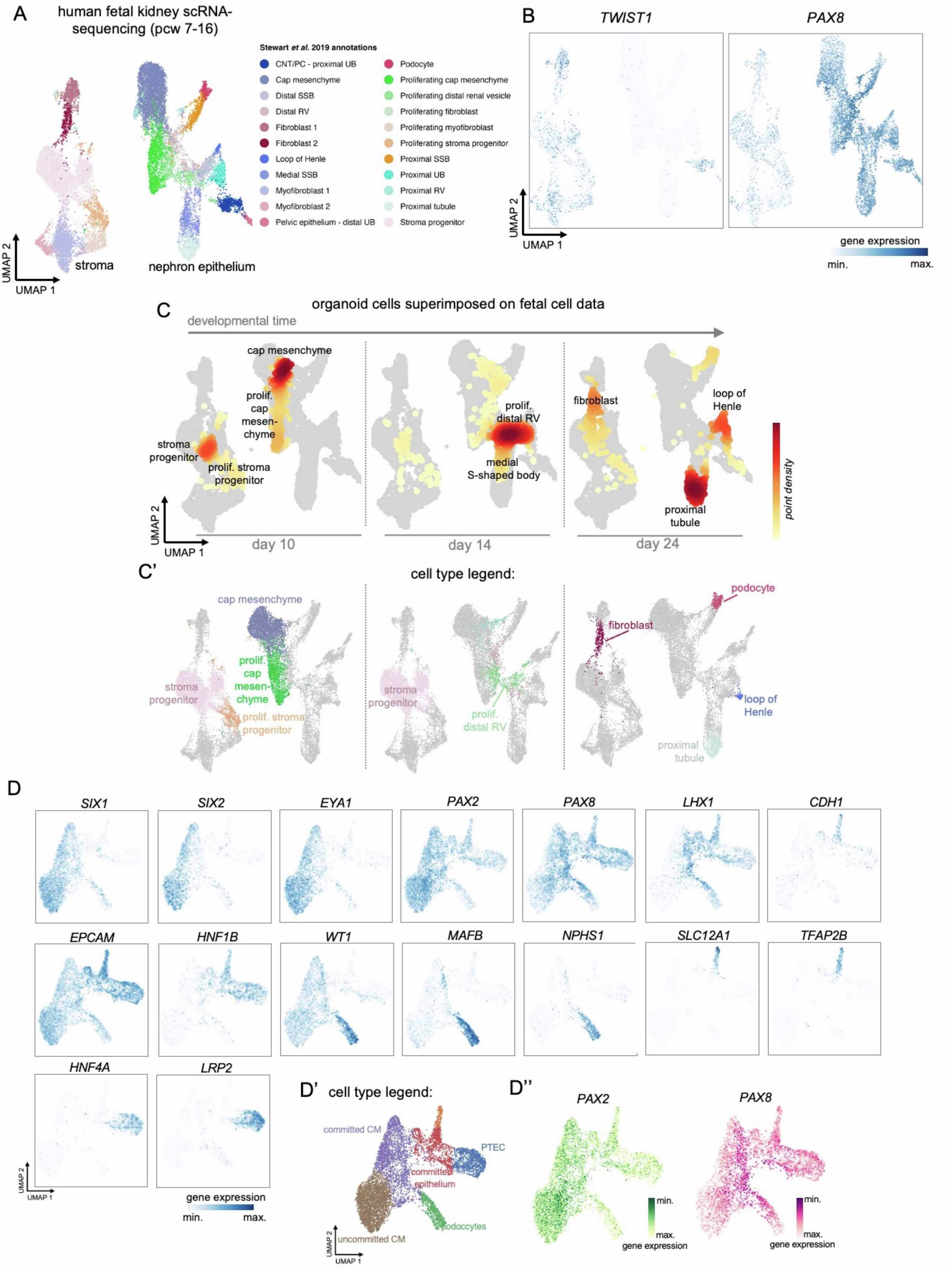

Ng-Blichfeldt et al_Supplemental Figure S2

Supplemental Figure S3, related to Figures 1 and 2. Clustering multi-ome cells reveals nephron cell types in day 14 epithelium

(A) UMAP plot of 9,147 multi-ome cells from batch 3 coloured by time point.

(B) UMAP plot of 9,147 multi-ome cells from batch 3 coloured by unsupervised clustering (resolution 0.1) and annotated according to time point and epithelial or stromal lineage.

(C) UMAP plot of 9,147 multi-ome cells from batch 3 coloured by unsupervised clustering (resolution 0.4).

(D) UMAP plot of 9,147 multi-ome cells from batch 3 coloured by unsupervised clustering (resolution 0.7).

(E) UMAP plot of 9,147 multi-ome cells from batch 3 coloured by unsupervised clustering (resolution 0.7), annotated according to time point, lineage and cell type as determined by marker genes identified from differential gene expression analysis (in E, F), with cells corresponding to C1 in (C) labelled as connecting tube.

(F) Heat map of expression of marker genes for clusters in (D) determined by snRNA-seq with representative markers annotated (left, log2FC > 1, FDR < 0.01, two-sided Wilcoxon rank-sum test; log2FC: log2 fold change; FDR: false discovery rate).

(G) UMAP plots of 9,147 multi-ome cells from batch 3 coloured by *WT1*, *HNF4A*, *TFAP2B* or *GATA3* gene expression.

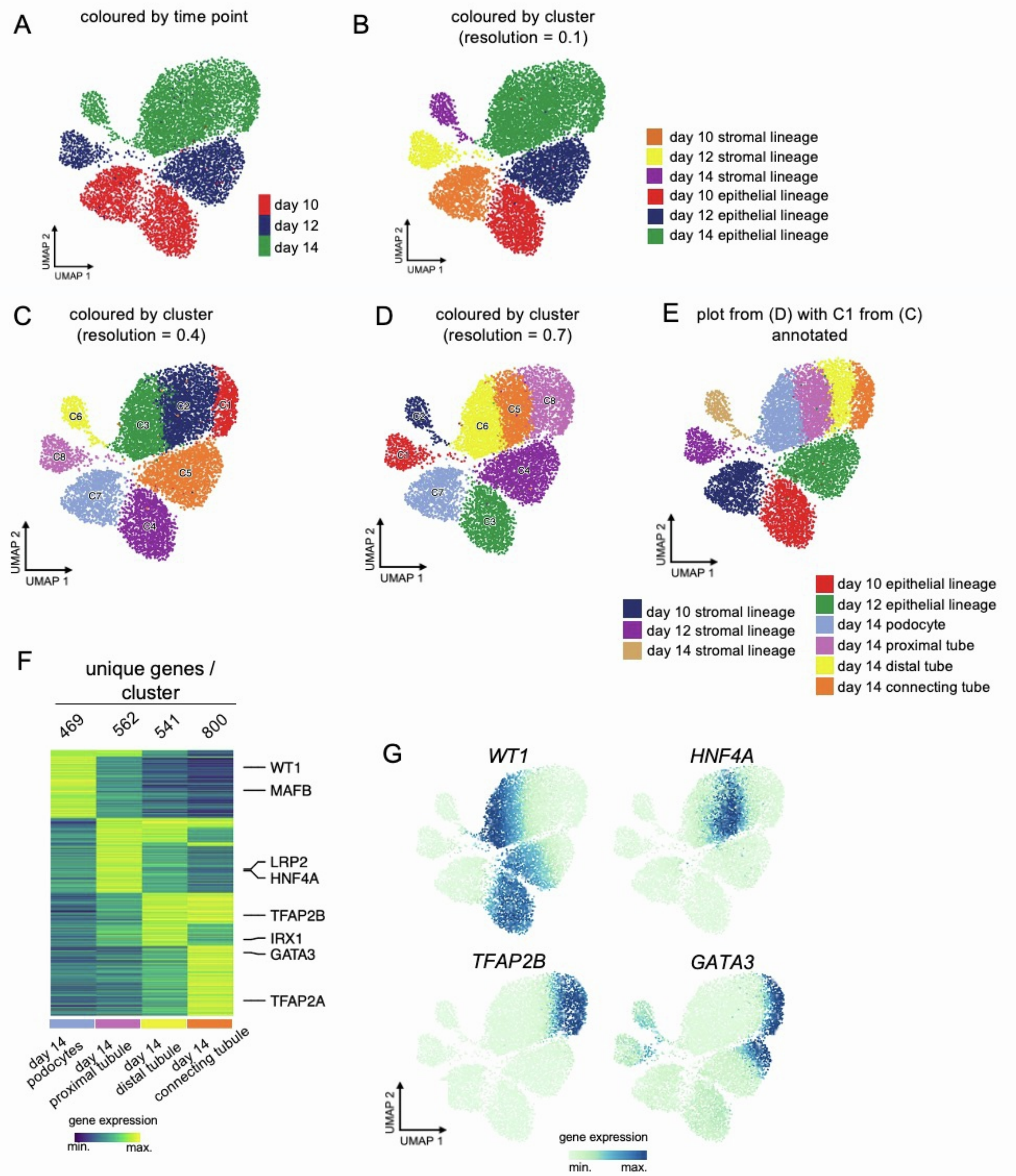

Ng-Blichfeldt et al_Supplemental Figure S3

Supplemental Figure S4, related to Figure 2. Analysis of an independent organoid multi-ome batch.

(A) UMAP of 3,040 cells from batch 2 clustered according to time point.

(B) UMAP of 3,040 cells from batch 2 clustered according to unsupervised clustering.

(C) Heat map of expression of marker genes for clusters in (A) determined by snRNA-seq with representative markers annotated (log2 FC > 1, FDR < 0.01, two-sided Wilcoxon rank-sum test).

(D) Heat map of marker peaks of accessible chromatin for clusters in (A) determined by snATAC-seq (left, log2 FC > 1, FDR < 0.01, two-sided Wilcoxon rank-sum test).

(E, E') Transcription factors plotted according to Pearson correlation coefficient (PCC) of gene expression versus corresponding motif accessibility, and log2FC gene expression in the epithelial compared to the stromal lineage as determined by snRNA-seq. Thresholds used to colour points according to principle outlined in (Fig. 2E): PCC > 0.2, Log2FC epithelial lineage > 1.5 (red = activators), PCC < -0.2, Log2FC epithelial lineage > 1.5 (blue = repressors).

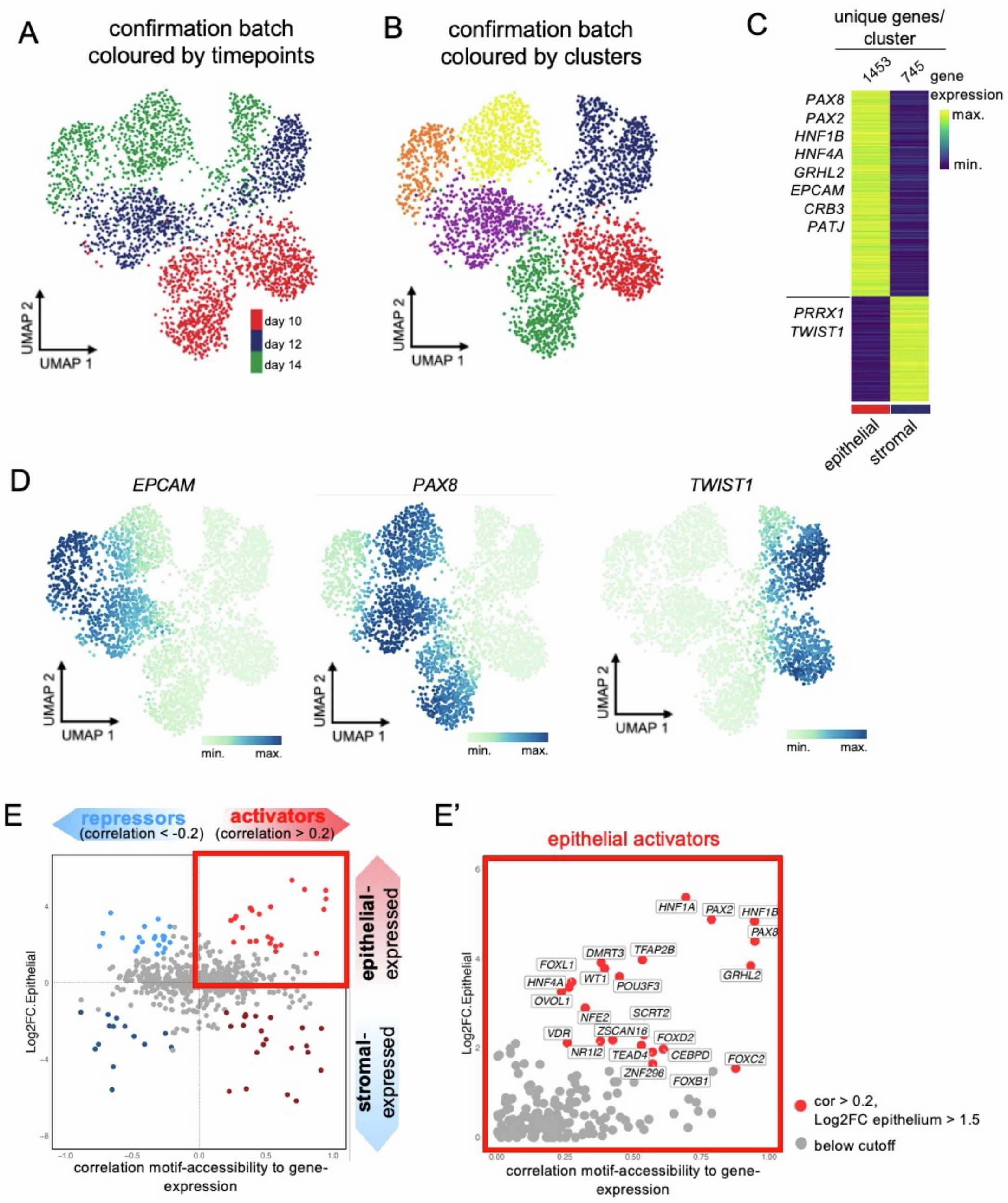

Ng-Blichfeldt et al_Supplemental Figure S4

Supplemental Figure S5, related to Figures 4 and 5. Expression of PAX8 and PAX2 in human renal organoid MET, and identifying the predicted PAX8 regulon.

(A) UMAP plots of 9,147 multi-ome cells from batch 3 showing gene expression (top) and motif accessibility (bottom) for PAX8 and PAX2, highlighting that PAX8 expression starts at day 10 and expression of PAX2 at day 12, but that accessibility of the shared motif is apparent at day 10.

(B) Immunofluorescence analysis of representative organoids at days 8, 10, 12 and 14, stained for PAX8 (top) or PAX2 (bottom), scale bars 25μm.

(C) Time course RT-qPCR data for *PAX8* and *PAX2* over the period of the kidney organoid protocol. Dots and bars represent mean ± SEM, N = 2-4 independent organoid batches at each time point.

(D) Immunofluorescence of organoids generated from a second independent PAX8-dCas9-KRAB iPSC line with a distinct gRNA to target PAX8 and harvested at day 14, either with no treatment (control, top) or following Dox treatment from day 4 (1μM Dox, bottom), showing GFP (green), PAX8 (magenta), E-Cadherin/CDH1 (yellow) with DAPI as counterstain (blue, nuclei). White brackets indicate $PAX8^{+}CDH1^{+}$ cells confined to a GFP^{-} region. Scale bars 25μm.

(E) Immunofluorescence of organoids treated with lentivirus inducing PAX8-overexpression (EF1α::PAX8/mCherry), showing mCherry expression in successfully transduced cells (magenta), anti-PAX8 (green) and TWIST1 (yellow) to label the stroma. Scale bars 30μm.

(F) Immunofluorescence of organoids generated from PAX2-dCas9-KRAB iPSCs (top 20% sorted, see STAR METHODS) and harvested at day 14, either with no treatment (control, top) or following Dox treatment from day 4 (1μM Dox, bottom), showing GFP (green), PAX2 (magenta), E-Cadherin/CDH1 (white) with DAPI as counterstain (blue, nuclei). Scale bars 25μm.

(G) RT-qPCR for *PAX2* in organoids generated from PAX2-dCas9-KRAB iPSCs (top 20% sorted, see STAR METHODS) and harvested at day 14, either with no treatment (control, white bars) or following Dox treatment from day 4 (1μM Dox, green bars). N = 3 independent organoid batches. Dots represent data from each batch normalised to corresponding control, with bars representing mean ± SEM. Unpaired t-test, **** ($p < 0.0001$), compared to control.

(H) Schematic of strategy used to identify peak to gene links, followed by motif analysis to identify predicted transcription factor target gene repertoires (related to Fig. 5).

(I) Violin plots of gene expression of *CDH1*, *CDH3, CDH4, CDH6* and *CDH16* in 9,147 multi-ome cells from batch 3 split by time point (related to Fig. 5).

(J) Immunofluorescence analysis of representative organoids at day 10 showing co-localisation of PAX8 (white) and CDH6 (green). Scale bars 20μm (related to Fig. 5).

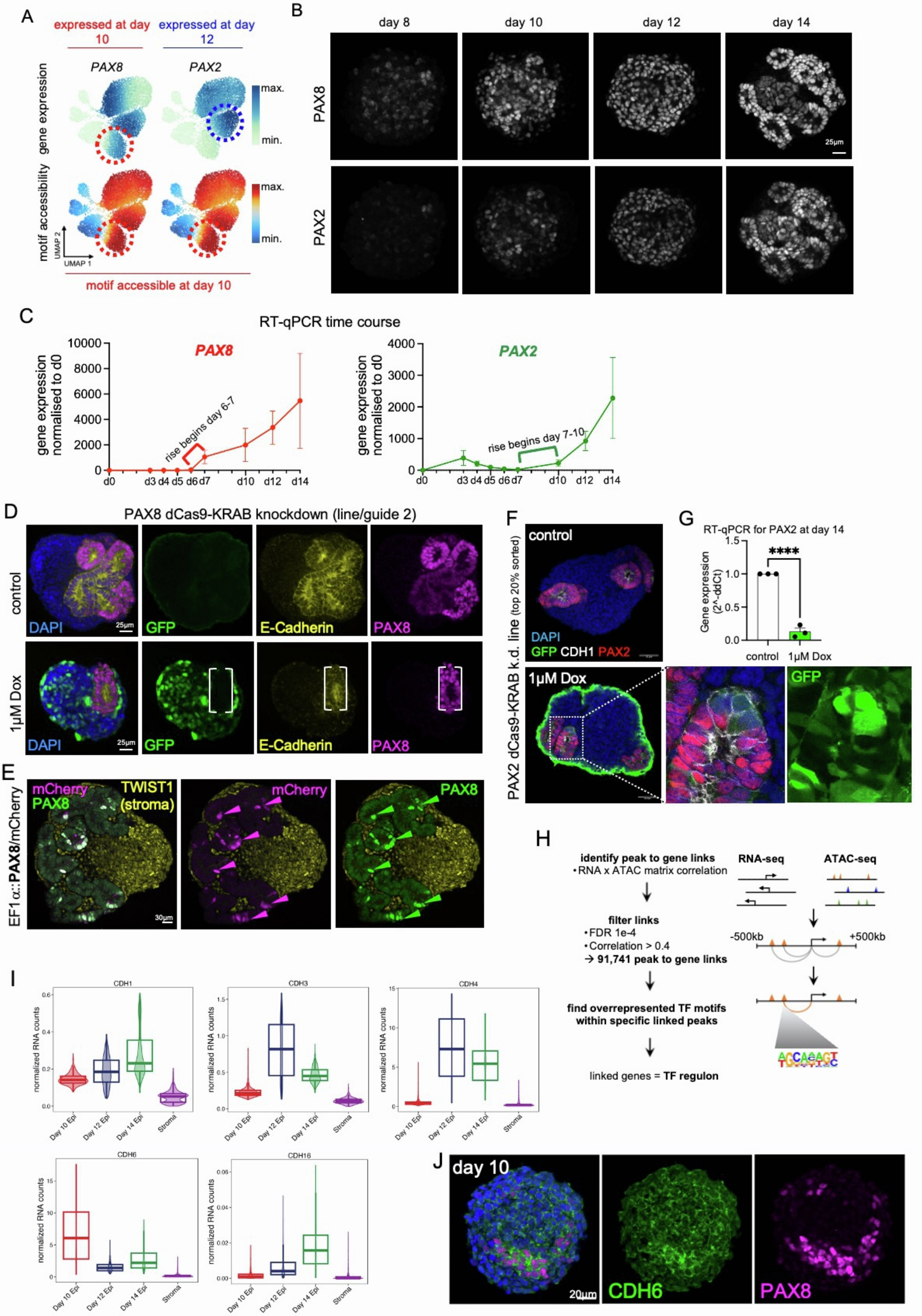

Ng-Blichfeldt et al_Supplemental Figure S5

Supplemental Figure S6, related to Figure 6. Transcription factor dynamics in an independent organoid multi-ome batch.

(A) UMAP plot of 3,040 cells from batch 2 coloured according to tube epithelial pseudotime trajectory, generated in ArchR (Granja et al., 2021).

(B) Cells in batch 2 plotted according to tube epithelial pseudotime as in (A), and gene expression (left) or motif accessibility (right), of the transcriptional activators *PAX8, PAX2* which show consistent motif accessibility along pseudotime, an example upregulated activators *HNF1B* and *HNF1A,* and example downregulated activators *LEF1* and *TCF7*.

(C) Cells in batch 2 plotted according to tube epithelial pseudotime as in (A), and gene expression (left) or motif accessibility (right), of example downregulated transcriptional repressors *SNAI2,* and *ZEB1,* and example upregulated repressor *FOXK1.*

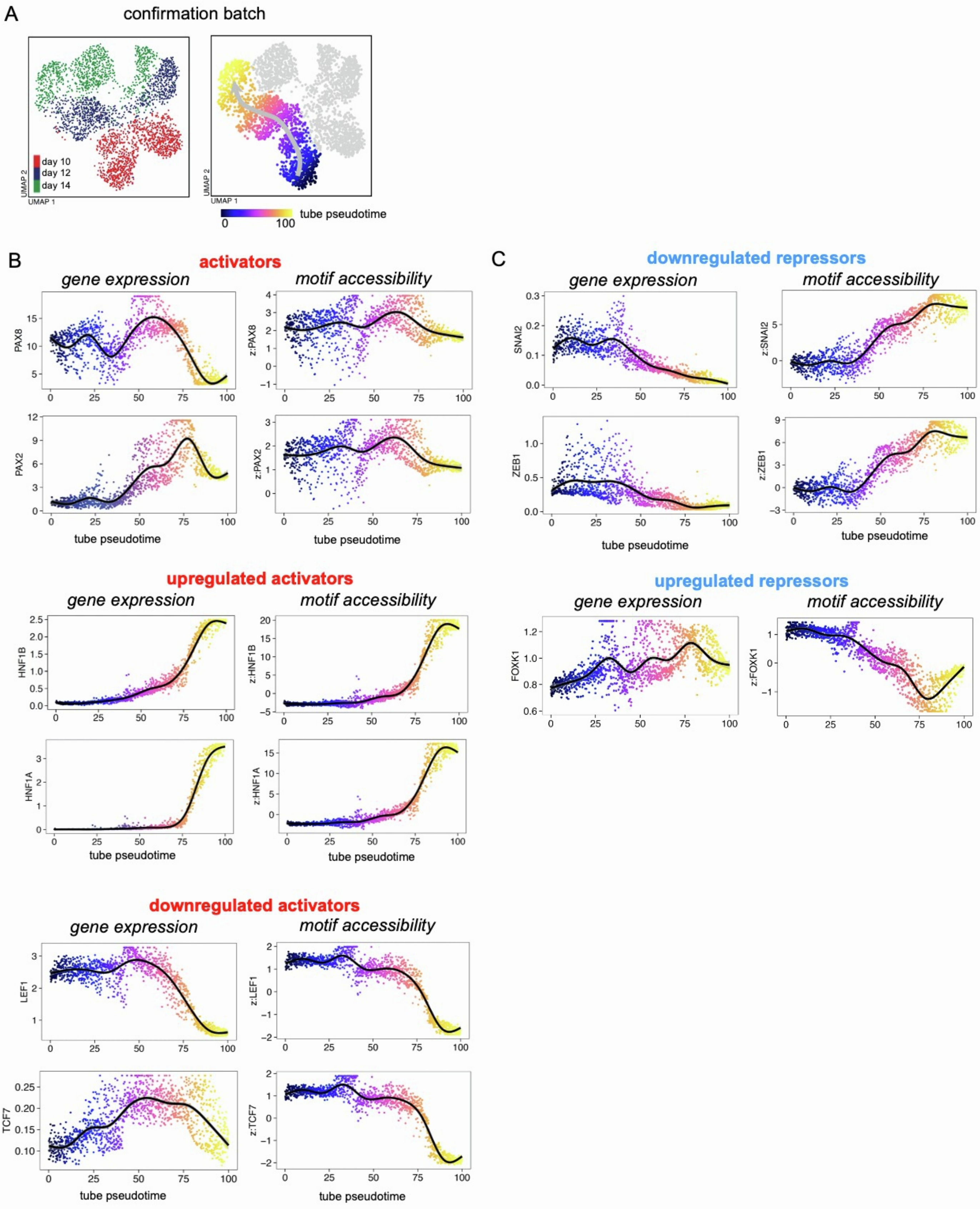

Ng-Blichfeldt et al_Supplemental Figure S6

Supplemental Figure S7, related to Figure 7. Effect of persistent Wnt/β-Catenin activation on MET in human renal organoids.

(A) Immunofluorescence of organoids at day 12 from same batch as shown in (B) and in Main Fig. 7B, showing expression of CDH1 (magenta), ZO1 (yellow) and HNF1B (green). Scale bars 20μm (top), 40μm (bottom).

(B) Insets from Fig. 7B of organoids treated with the indicated conditions between days 12 to 14, showing individual channels of E-Cadherin/CDH1 (magenta), ZO1 (yellow) and HNF1B (green). Scale bars 20μm.

(C) Schematic of the experimental set-up to test the reversibility of the effect of sustained Wnt/β-Catenin signalling with CHIR washout between days 14 and 17 by immunofluorescence as shown in panel (D).

(D) Organoids harvested at day 17 following treatments indicated in (C), with light microscopy images (top, scale bar 400μm), and immunofluorescence images showing expression of HNF1B (green), E-Cadherin/CDH1 (magenta) and ZO1 (yellow). Dotted white boxes indicate positions of magnification panels below. Scale bars 50μm.

(E) Schematic of the overall renal organoid multi-ome profiling strategy for batch 3 – corresponding to the schematic in Fig. 1D but including the two day 14 samples sequenced in parallel: one treated with DMSO from day 12 to 14 (analogous to normal protocol), and one treated with 3μM CHIR from day 12 to 14.

(F) UMAP plot of 13,502 multi-ome cells from batch 3, of organoids harvested at day 10, day 12, and at day 14 treated either with DMSO or 3μM CHIR from day 12 to 14 (See Fig. 1D-E), coloured according to sample.

(G) UMAP plots of 13,502 multi-ome cells from batch 3 as in (F), coloured according to gene expression of *PAX8* (left), *EPCAM* (centre), or *TWIST1* (right).

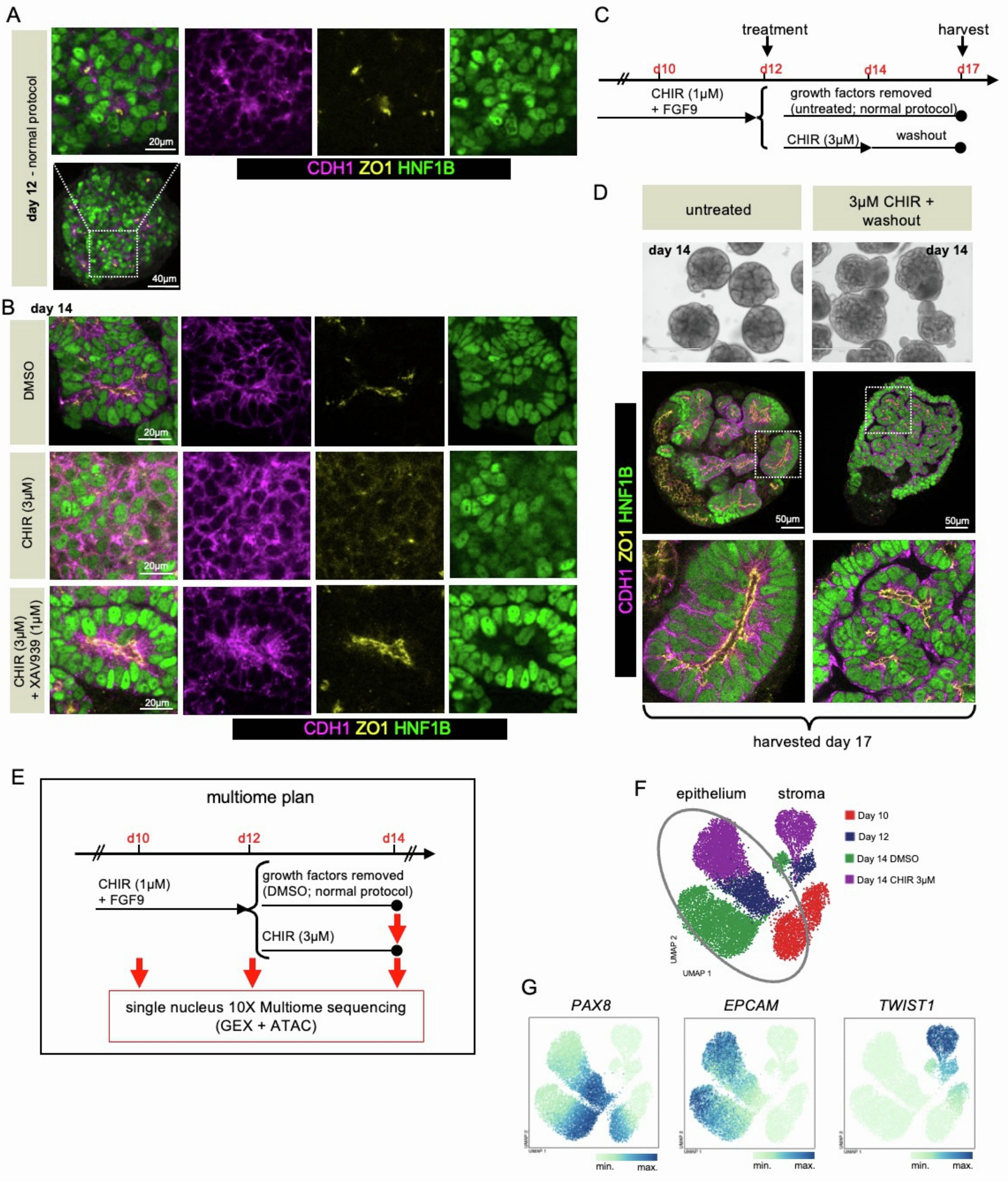

Ng-Blichfeldt et al_Supplemental Figure S7

Supplemental Figure S8, related to Figure 7. Persistent Wnt/β-Catenin activation in human renal organoids distalises the epithelium via inhibition of Notch signalling.

(A) UMAP plots of 10,793 multi-ome cells from batch 3 (as in Main Fig. 7C) showing persistent Wnt pathway activation in day 14 cells treated with 3μM CHIR from day 12 to 14 compared to day 14 cells cultured according to the normal protocol, as shown by retained *AXIN2* gene expression and TCF7 motif accessibility.

(B) UMAP plots of 10,793 multi-ome cells from batch 3 (as in Main Fig. 7C) showing continued expression of day 12 tubule marker genes (*LHX1, UNCX*) in day 14 cells treated with 3μM CHIR from day 12 to 14 compared to day 14 cells cultured according to the normal protocol where their expression is normally downregulated.

(C) Gene ontology analysis of genes enriched in day 14 epithelium treated with DMSO from day 12-14 compared to 3μM CHIR, showing top 5 highest specific GO Molecular Function terms by log10 adjusted p value, full list in Supplemental Table 7.

(D) UMAP plots of 10,793 multi-ome cells from batch 3 (as in Main Fig. 7C) showing altered gene expression of *WT1, HNF4A,* and *GATA3* in day 14 cells treated with 3μM CHIR from day 12 to 14 (highlighted by dotted circle) compared to day 14 cells cultured according to the normal protocol.

(E) UMAP plots of multi-ome cells from batch 3 (as in Main Fig. 7C) showing altered motif accessibility of WT1, HNF4A, and TFAP2B in day 14 cells treated with 3μM CHIR from day 12 to 14 (highlighted by dotted circle) compared to day 14 cells cultured according to the normal protocol.

(F) UMAP plot of 7,871 multi-ome cells from batch 3 comprising only day 14 cells treated either with DMSO or 3μM CHIR from day 12 to 14, subjected to unsupervised clustering and coloured according to nephron segment (in DMSO condition). The CHIR-treated epithelium showed 2 clusters by unsupervised clustering.

(G) Heatmap of motif accessibility of selected transcription factors in cell clusters as in (F). Accessibility of TCF7, LEF1, EHF and ELF3 motifs is increased in day 14 epithelium treated with 3μM CHIR from day 12 to 14, consistent with sustained Wnt/β-Catenin activity. Accessibility of RBPJ motifs, and of WT1 and HNF4A motifs, are lost in CHIR-treated epithelium, consistent with inhibition of Notch signalling and a loss of proximal identity, respectively. HNF1B and GRHL2 motifs are greatly reduced in CHIR-treated epithelium.

(H) UMAP plots of multi-ome cells from batch 3 (as in F) showing altered motif accessibility of RBPJ, LEF1 and TCF7 in day 14 cells treated with 3μM CHIR from day 12 to 14 compared to day 14 cells cultured according to the normal protocol.

(I) Violin plots of multi-ome cells from batch 3 (as in F) showing reduced gene expression of the Notch pathway receptor *NOTCH2* and of the Notch ligand *DLL1* expression in CHIR-

treated cells at day 14 compared to day 14 cells cultured according to the normal protocol. Box centre line, median; limits, upper and lower quartiles; whiskers, 1.5x interquartile range.

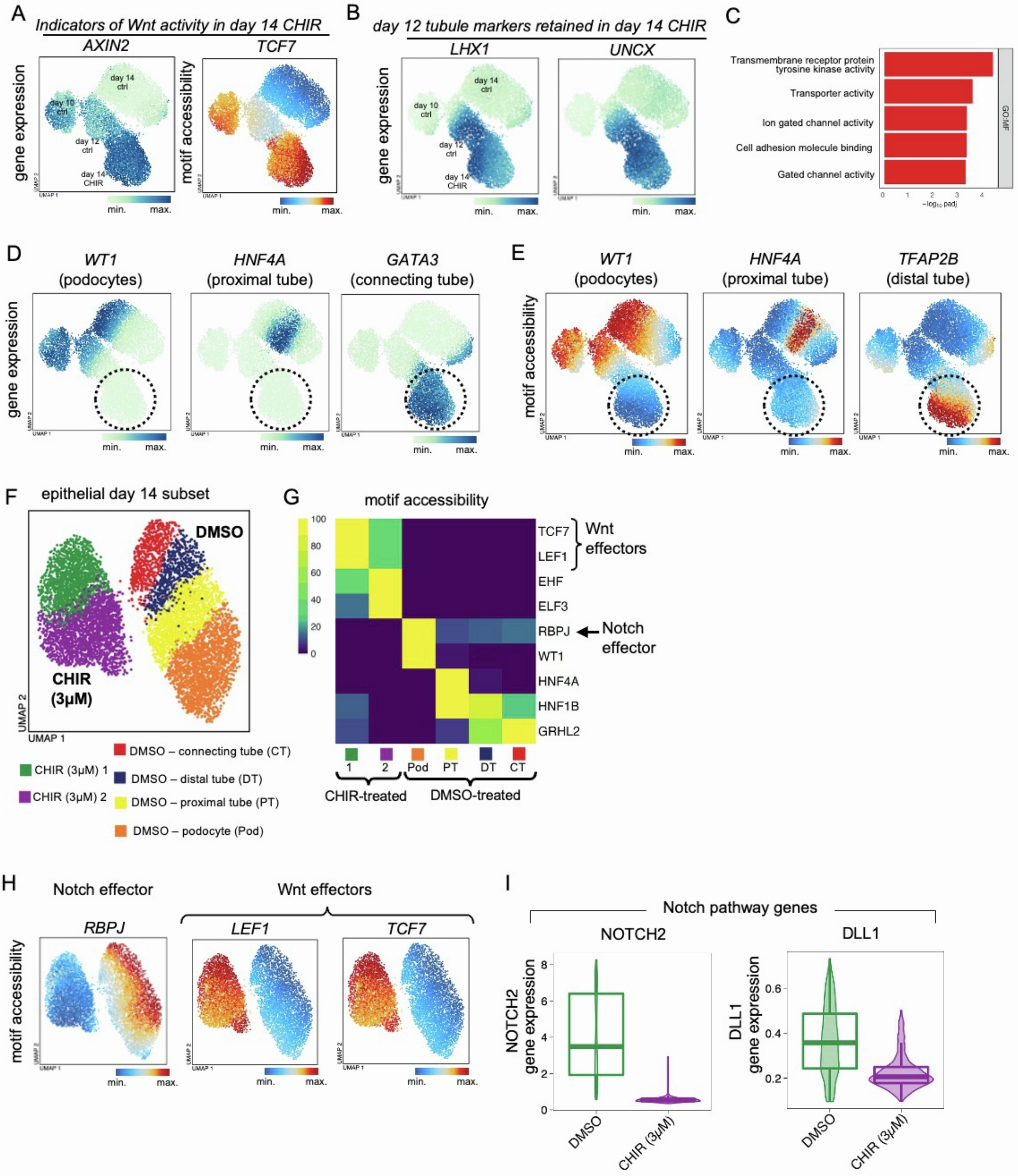

Ng-Blichfeldt et al_Supplemental Figure S8
